# Cross-Linking Strategies for Fluorine-Containing Polymer Coatings for Durable Resistant Water- and Oil-Repellency

**DOI:** 10.3390/polym13050723

**Published:** 2021-02-27

**Authors:** Julia Kredel, Deborah Schmitt, Jan-Lukas Schäfer, Markus Biesalski, Markus Gallei

**Affiliations:** 1 Polymer Chemistry, Universität des Saarlandes, Campus Saarbrücken, 66123 Saarbrücken, Germany; j.kredel@mc.tu-darmstadt.de (J.K.); deborah.schmitt@uni-saarland.de (D.S.); 2 Ernst-Berl Institute of Technical and Macromolecular Chemistry, Technische Universität Darmstadt, Alarich-Weiss-Straße 4, 64287 Darmstadt, Germany; j.schaefer@cellulose.tu-darmstadt.de (J.-L.S.); biesalski@tu-darmstadt.de (M.B.)

**Keywords:** cross-linking reactions, emulsion polymerization, fluorine-containing polymers, oil- and water repellency, durable-resistance

## Abstract

Functional coatings for application on surfaces are of growing interest. Especially in the textile industry, durable water and oil repellent finishes are of special demand for implementation in the outdoor sector, but also as safety-protection clothes against oil or chemicals. Such oil and chemical repellent textiles can be achieved by coating surfaces with fluoropolymers. As many concerns exist regarding (per)fluorinated polymers due to their high persistence and accumulation capacity in the environment, a durable and resistant coating is essential also during the washing processes of textiles. Within the present study, different strategies are examined for a durable resistant cross-linking of a novel fluoropolymer on the surface of fibers. The monomer 2-((1,1,2-trifluoro-2-(perfluoropropoxy)ethyl)thio)ethyl acrylate, whose fluorinated side-chain is degradable by treatment with ozone, was used for this purpose. The polymers were synthesized via free radical polymerization in emulsion, and different amounts of cross-linking reagents were copolymerized. The final polymer dispersions were applied to cellulose fibers and the cross-linking was induced thermally or by irradiation with UV-light. In order to investigate the cross-linking efficiency, tensile elongation studies were carried out. In addition, multiple washing processes of the fibers were performed and the polymer loss during washing, as well as the effects on oil and water repellency were investigated. The cross-linking strategy paves the way to a durable fluoropolymer-based functional coating and the polymers are expected to provide a promising and sustainable alternative to functional coatings.

## 1. Introduction

In recent years, functional surfaces have been of growing importance within research strategies leading to more functional and sustainable materials. An established strategy to tailor surfaces of materials and to provide certain functionalities is the application of a coating. In this way, surfaces have been modified with functionalities that serve as protection against scratches, graffiti, and other contamination [1,2], as well as providing improved UV-resistance or fire resistance [3,4]. Thus, with the use of a thin layer of a feasible surface coating, long-term stability can be achieved along with additional useful features in addition to the bulk materials’ properties. Potential further applications are wide-spread and are in fields of biomedicine, paints, and varnishes [5] for leather [6], as a coating for textiles [7] with an anti-sticking [8,9], anticontamination [10,11], antifouling, or a self-cleaning effect [1,2]. Especially, the textile industry is a sector in which surface finishing renders a continuously growing demand [12]. There is a growing requirement for functional materials for increased mechanical stress, but above all for textiles in the outdoor sector with increased water repellency. Already Neinhius and Barthlott have been inspired by nature in order to generate a durable water repellency by imitating the surface of a lotus leaf, which features a hierarchical fine structuring on the nanometer and micrometer length scale, combined with a coating of a hydrophobic wax [11,13]. In this way, super-hydrophobicity is achieved in nature also in rice and rose leaves, butterfly wings, and gecko feet [14,15,16,17]. Artificial superhydrophobic surfaces were accessible using surface roughening in combination with a hydrophobic coating [1,18,19,20]. These surface modifications became possible by coating with hydrophobic polymer material [21,22] but also by using nonpolar dendrimers or patterned nanoparticles [23]. With respect to water-repellent textiles, the latter are often used to increase comfort, e.g., in sportswear with rain protection, where sweat can permeate from the inside to the outside. On the other side, rain cannot penetrate from the outside to the inside [18,24]. Thus, depending on the chemistry of the coatings used, not only hydrophobicity but also increased oil repellency, chemical repellency, or dirt repellency can be achieved, e.g., for use in workwear [22]. For this purpose, in general, the surface energy of the substrate must be reduced to a level below the surface tension of the respective liquids [25]. For the wetting with water, this can be achieved for instance by the application of long-chain hydrocarbons. For oil repellency, a coating based on fluoropolymers is still the most suitable method [26]. Polymers with perfluorinated groups feature surface characteristics that are quite different from those of non-fluorinated materials. In addition to excellent chemical and thermal stability [27], they also show a low refractive index [28], non-adhesive properties [29], and a rather low surface energy [30]. In general, fluorine-containing polymers were designed for use in space missions precisely as a result of their exceptional properties, but they were able to revolutionize the market for functional textiles at an early stage. Due to their extremely low surface energies, fluoropolymers are suitable both for water repellency and oil repellency [31,32]. Thus, surface energies as low as 6 mN/m and a very high contact angle can be generated on surfaces with the tightest packing of CF_3_ end-groups [27,33]. Since fluoropolymers show neither affinity for water nor for oil and tend to form miscibility gaps, the terminology fluorophilic was introduced [26]. While the most common fluoropolymer is still PTFE, the side-chain fluorinated polymers based on fluorotelomers are used for the coating of textiles or other surfaces [26]. There are currently over 4700 different side-chain fluorinated perfluoralkyl substances on the market [34], most of which form PFOS and PFOA derivatives by the degradation of the side-chain of the polymer and are released into the environment [35,36]. Due to their enormous stability and inertness, these PFOS derivatives are bioaccumulative in the environment, as well as in the human organism, and in some cases, these compounds are stable for years [37,38]. Although no real toxic effect has been proven yet, it is alarming to see the accumulation of these substances in all food chains of the world, as well as in the blood or often in the liver of humans and animals [39]. Despite unknown effects in the body, increasing liver cancer and immune deficiency occur in contaminated individuals. Moreover, half-life times of fluorine compounds in the blood of up to eight years have been found [40,41]. In recent years, awareness of the hazardous effects of long-chain perfluorinated and persistent chemicals has increased, leading to their inclusion in guidelines such as the Stockholm Convention, on the list of persistent organic pollutants (POP), or in voluntary stewardship programs [42,43]. Thus, the trend goes towards less environmentally hazardous materials in recent years [44]. In addition, the degradation of highly fluorinated coating materials has already been investigated by high-energy irradiation or ultrasonication, but this degradation requires enormous amounts of energy and is more suitable for low-level contamination [45]. Thermal degradation could also show limited effects, but requires very high temperatures (PTFE degradation temperature: 600 to 800 °C [46]) and still leads to perfluorinated derivatives as degradation byproducts [47]. Current research on perfluoropropylvinylether (PPVE), or a monomer based on this PPVE (2-((1,1,2-trifluoro-2-(perfluoropropoxy)ethyl)thio)ethyl acrylate) with ether and thioether moieties as linkers in the fluorinated side-chain has already yielded promising results with respect to the degradation of the polymer in the presence of ozone in the atmosphere [32,48]. In this case, the maximum length of the fluorinated fragments are perfluoropropyl-segments, while low-molecular fluorophosgenes, which are harmless in the atmosphere are also formed [48]. Nevertheless, the diffuse release of fluoropolymers during the coating process, the use and exposure of the coated textiles, and especially the washing processes of the textiles must be prevented, which releases them into the environment via wastewater, sometimes without purification. Consequently, the durability of the coating, even during washing and exposure is highly demanding, yet not always trivial using existing technologies. Hence, it is necessary to further develop cross-linking strategies for durable hydrophobic and oleophobic coatings based on fluoropolymers like PPVE. Therefore, different cross-linking strategies for fluorine-containing polymers will be investigated. A well-established method for cross-linking of fluoropolymers is the irradiation of P(TFE-*co*-HFP) with high-energy light, as described in the review by Lyons et al., so that the copolymer forms a strong mesh [49]. To achieve a chemically robust and more resistant coating on surfaces, textiles, or fibers, it is necessary to create a covalent bond with the fibers themselves. For this purpose, a thermal cross-linking reaction based on diisocyanates is investigated here with the aid of hydroxyl groups in the polymer cross-link the fibers by means of a urethane bond [50]. The second thermal cross-linking strategy investigated here is cross-linking by introducing an epoxide group into the polymer that connects with the hydroxyl groups of the fibers by a ring-opening reaction [51]. The last cross-linking strategy based on a UV cross-linker is realized by introducing a benzophenone monomer into the polymer. Irradiation with UV light will lead to cross-linking by proton abstraction [52].

Within this contribution, a copolymer consisting of 2-((1,1,2-trifluoro-2-(perfluoropropoxy)ethyl)thio)ethylacrylate (so-called fluoroacrylate (FA)) and stearyl methacrylate is synthesized by emulsion polymerization, incorporating the three different thermal, as well as UV-induced, cross-linking strategies. In addition, different amounts of cross-linking reagents are incorporated into the polymer to evaluate the effectiveness of these strategies. Subsequently, the different polymers are analyzed by NMR and DSC, applied to cellulose fibers, and subsequently cross-linked. The complete and durable cross-linking is demonstrated and discussed by results obtained from tensile-strain measurements, solvent treatment, and confocal microscopy measurements, and the substrates are examined with respect to their water repellency using the static contact angle and for their oil repellency using the hydrocarbon resistance test. Finally, using repeated washing steps, the mechanical integrity of the polymeric fiber coatings is investigated. Based on these studies, a feasible and effective cross-linking strategy for the preparation of durable oil- and water-repellent coatings based on fluoropolymers will be investigated, paving the way to a more environmentally friendly fluorine-containing polymer coating in the textile industry.

## 2. Experimental Section

### 2.1. Materials

All solvents and reagents were purchased from Fisher Scientific (Schwerte, Germany), Sigma-Aldrich (St. Louis, MA, USA) TCI (Eschborn, Germany), ABCR (Karlsruhe, Germany) and used as received unless otherwise stated. The cross-linking reagent hydroxyethyl methacrylate (HEMA) and glycidyl methacrylate (GlyMA) were purchased from Fisher Scientific and passed through an alumina column (basic, 50 to 200 µm, Acros Organics) prior to use. The 3-hydroxy benzophenone methacrylate (BPMA) was obtained from Alfa Aesar (by Thermo Fisher Scientific) and used without further purification. Deuterated solvents were purchased from Sigma-Aldrich. The monomer 2-((1,1,2-trifluoro-2-(perfluoropropoxy)ethyl)thio)ethylacrylate (FA) was provided by Merck KGaA (Darmstadt, Germany) and distilled under reduced pressure. To remove the inhibitor of stearyl methacrylate monomer (StMA), the solid monomer was melted at 50 °C, diluted with THF (extra pure) to a final content of 50% THF and 50% monomer, and passed through an alumina column (basic, 50 to 200 µm, Acros Organics) followed by distillation of the solvent. Prior to the use for polymerization, stearyl methacrylate was liquefied at 50 °C. Rhodamine B-methacrylamide (RhBMA) was synthesized as described in the supporting information and the literature [53,54]. Commercially available filter discs (grade 3 hw, Munktell, Ahlstrom, Stockholm, Sweden) were used as model cellulose substrates featuring diameters of 90 and 180 mm and a grammage of 65 g/m^2^. The paper discs consist of bleached α-cellulose consisting of pine and spruce fibers featuring median pore sizes of 8 to 12 µm. Polyamide textile for SEM measurements were donated from TransTextile (Freilassing, Germany).

### 2.2. Instrumentation

Standard size exclusion chromatography (SEC) was performed with a system composed of a 1260 IsoPump-G1310B (Agilent Technologies, Santa Clara, CA, USA), a 1260 RI detector (G2362A) at 30 °C (Agilent Technologies), and a 1260 VW detector (G1314F) at 254 nm (Agilent Technologies). THF as the mobile phase with a flow rate of 1 mL/min was used on an SDV column set from polymer standard service (PSS, Mainz, Germany) (SDV 10^3^, SDV 10^5^, SDV 10^6^). The calibration was carried out using PS standards (from PSS). For data acquisition and evaluation of the measurements, PSS WinGPC UniChrom 8.2 was used. ^1^H-NMR spectra were recorded on a Bruker DRX 300 spectrometer (Billerica, MA, USA) working at 300 MHz. The NMR chemical shifts are referenced relative to the used deuterated solvents. The ^1^H-NMR spectroscopy samples of the polymer dispersion were separated from water by freeze-drying and for analyzing 20 mg of polymer was dissolved in 0.55 mL hexafluorobenzene and 0.05 mL CDCl_3_. For determination of the thermal properties of the synthesized polymers, differential scanning calorimetry (DSC) was performed with a Mettler Toledo DSC^−1^ (Columbus, OH, USA) in a temperature range of −100 to 200 °C with a heating rate of 20 K/min under a nitrogen atmosphere. IR spectroscopy was performed on a Spectrum One instrument from PerkinElmer (Waltham, MS, USA) in attenuated total reflection mode (ATR). The IR spectra were recorded from 4000 to 400 cm^−1^. The cellulose samples were coated with the polymer dispersions in a Mathis two-roll laboratory foulard, horizontal type HF (Mathis GmbH, Oberhasli, Switzerland) at a rotation rate of 2 m/min and a contact pressure of 3 bar. Scanning electron microscopy measurements (SEM) were conducted on a Philips XL 30FEG (Philips, Amsterdam, Netherlands) with an operating voltage of 15 kV. The samples were coated with a 10 nm thick layer of Pd/Pt (80/20), using a Quorum Q300T D sputter coater (Lewes, UK). The static contact angle (CA) was measured using the sessile-drop method with a Contact Angle System DataPhysics OCA 115 EC (Filderstadt, Germany) using 4 µL droplets of deionized water. The measurements were conducted in a controlled climatic chamber at T = 23 ± 2 °C and a relative humidity of 50%. The contact angles were determined geometrically using the SCA20 software by aligning a tangent from the surficial contact point along the droplets surface in the droplet profile. The oil repellency was determined with the hydrocarbon test for oil repellency according to the American Association of Textile Chemists and Colorists AATCC test method 118 with four hydrocarbon test liquids (*n*-tetradecane, *n*-dodecane, *n*-decane, and *n*-octane) as described in the literature [32,55,56]. For the complete thermal cross-linking reaction of the polymer with the cellulose fiber, the coated samples were treated in a VTR 5022 oven (Heraeus, Hanau, Germany) at 120 or 160 °C under reduced pressure for at least 4 h. For the UV-cross-linking reaction, a UVA-Cube 2000 (Dr. Hoenle AG, Gräfelfing, Germany) was used and the samples were irradiated with a mercury lamp and an output power of 1000 W for 5 min at each side of the sample. The tensile strength was determined on a ZwickRoell Z1.0 with a 1 kN X-force P load cell using the software testXpert II V3.71 (ZwickRoell, Ulm, Germany) at a tensile speed of 10 mm/min. The samples were stored for 24 h in a climate chamber at T = 23 ± 2 °C and a humidity of 50% and also measured there. Brightfield and confocal fluorescent images were recorded on a TCS SP8 Confocal Laser Scanning Microscope (CLSM, Leica Microsystems GmbH, Mannheim, Germany) with an HCX PL APO20xNA 0.7 Immor HCX PL APO 63xNA 1.2 W CORR objective. Brightfield images were obtained using a 488 nm laser for illumination and detecting the transmitted light on a photomultiplier tube (PMT). Fluorescent samples were imaged by sequentially exciting each pixel line of the confocal scan with 405 and 552 nm lasers, corresponding to the excitation wavelengths of Calcofluor White and rhodamine B methacrylamide. Emission was detected between 420 and 470 nm for Calcofluor White and 575 and 630 nm for RhBMA labeled polymer on a sensitive LeicaHYD detector and a PMT detector.

### 2.3. Exemplary Synthesis of Poly(fluoroacrylate-co-Stearyl Methacrylate) via Emulsion Polymerization

The poly(fluoroacrylate-*co*-stearyl methacrylate) (P(FA-*co*-StMA)) was synthesized according to starved feed emulsion polymerization protocols [32]. A 250 mL double-walled reactor, equipped with a reflux condenser and a stirrer, was heated to 80 °C and purged with a steady stream of nitrogen. The reactor was filled with a dispersion of 1.86 g (4.6 mmol) fluoroacrylate, 1.24 g (3.6 mmol) stearyl methacrylate, 37 mg (1.28 mmol) sodium dodecyl sulfate (SDS), 10 g 1,2-propanediol, and 90 g of degassed and deionized water. The polymerization was initiated by adding solutions of 20 mg (0.1 mmol) sodium metabisulfite (SBS) in 2 g water and 200 mg (0.84 mmol) sodium peroxodisulfate (SPS) in 2 g water. After 10 min, a monomer emulsion (ME) consisting of 150 mg (0.52 mmol) SDS, 18.7 g (46.9 mmol) fluoroacrylate (FA), 12.3 g (36.4 mmol) stearyl meth acrylate, 3.75 g 1,2-propanediol, and 33.5 g water was dosed continuously with a flow rate of 0.5 mL/min by a rotary piston pump. After complete dosing of the monomer emulsion, the reaction mixture was kept at 80 °C for another hour. The finished polymer dispersion is cooled, filled, and stored overnight in a refrigerator at 8 °C, so the unreacted stearyl methacrylate monomer solidifies and can be separated by filtration. The polymer dispersion is milky white and has a solid content of 13.8 wt% polymer in water.

### 2.4. Application of Polymer-Dispersion on Paper Substrates

To achieve a comparable polymer content on the fiber for all different polymer dispersions, the solid content of the dispersion was reduced with deionized water to 5 wt%. A total of 50 mL of the polymer dispersion was filled into the laboratory foulard. The cylinders of the foulard were pressed against each other with a pressure of 3 bar and rotated with a speed of 2 m/min. The cellulose fibers were passed twice through the foulard, to ensure the complete wetting of the fiber. After coating, the substrates were first air-dried at room temperature and subsequently annealed in an oven at 90 °C for one hour. For substrates to be thermally cross-linked with the aid of diisocyanate, in a second step a solution of the diisocyanate in dichloromethane (DCM) was added into the foulard and the substrates already coated with polymer were again coated with this solution. All substrates were weighted prior to coating, directly after coating under wet conditions, and after annealing in dry conditions to calculate dispersion loading and polymer absorption. Depending on the cross-linking strategy, the coated substrates were cross-linked at 120 °C or 160 °C in the oven under reduced pressure (600 mbar) or cross-linked by UV radiation using a UVA cube.

### 2.5. Model Experiment to Investigate the Chemical Stability

In order to verify whether the cross-linking of polymers on the fibers by cross-linking strategies presented here was successful and whether a chemical integrity can be achieved, a continuous intensive treatment with solvent was performed. For this purpose, the polymer-coated and cross-linked cellulose fibers were extracted with tetrahydrofuran (THF) in a Soxhlet apparatus for 24 h to simulate a multiple-wash process. Since the polymer is not soluble in water, THF was used for extraction to increase the strain on the cross-linking on the fiber. In this case, mechanical stress was not involved. Mechanical friction could increase the stress on the coating even more, resulting in significant deviations in the results.

The cellulose substrates were weighed before and after coating and after extraction in order to calculate the mass loss due to dissolved polymer.

## 3. Results and Discussion

In the following, a highly fluorinated and ozone-degradable polymer for application on cellulose fibers for an oil- and water-repellency will be synthesized according to previously described protocols [32]. In this study, filter paper discs were used as model substrates of cellulose to evaluate the chemical modifications. For investigating the durable surface modification, three different cross-linking strategies for the fluorinated copolymers on cellulose fiber substrates are presented and evaluated in order to obtain an ideal system for the formulation of durable resistant coatings. We investigated two thermal and one UV-mediated cross-linking strategies for the fluorine-containing polymers. Within the following four sections, the different polymers are synthesized and characterized and application of the polymers onto cellulose fibers are described. As a focus, the cross-linked polymers immobilized at surfaces were characterized and the chemical integrity of the coatings was evaluated.

### 3.1. Synthesis and Characterization of Poly(fluoroacrylate-co-Stearyl Methacrylate) P(FA-co-StMA) with Cross-Linking Reagents

**Figure 1:**
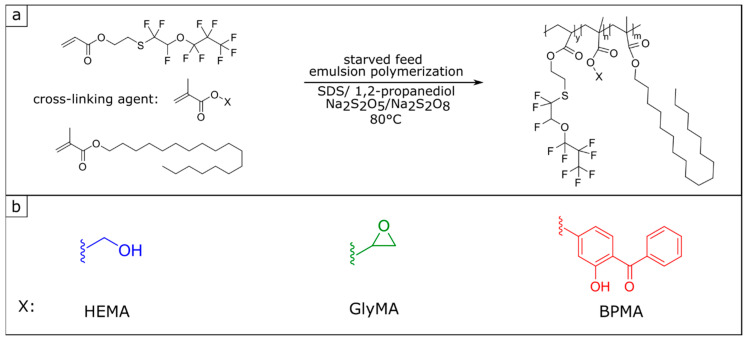
(**a**) Reaction scheme of the starved feed emulsion polymerization of fluoroacrylate (FA), stearyl methacrylate (StMA), and the cross-linking agent X, (**b**) shows the cross-linking reagents hydroxyethyl meth acrylate (HEMA), glycidyl methacrylate (GlyMA), and 3-hydroxy benzophenone methacrylate(BPMA).

For polymer synthesis, a combination of the monomer 2-((1,1,2-trifluoro-2-(perfluoropropoxy)-ethyl)thio)ethyl acrylate (FA) and stearyl meth acrylate (StMA) was chosen and polymerized by means of semi-continuous starved feed emulsion polymerization. The use of StMA is known to additionally support the water-repellent effect because of its comparatively low surface energy (33.2 mN/m for StMA [57]). It is worthy to mention that the emulsion polymerization leads to a polymer dispersion in water, which can be directly used for the application as a surface coating and the functional polymer can be applied immediately without further purification. To promote the diffusion of the highly water-insoluble monomers, the co-solvent 1,2-propanediol is added already during the emulsion polymerization [20,58]. The slow dosing rate during the starved feed emulsion polymerization (0.5 mL/min) provides an immediate reaction of the monomers, which also ensures a statistical distribution of the monomers in the polymer, despite different copolymerization parameters. The polymer dispersions containing cross-linking components are prepared according to the same protocol as described in Section 2.3 and shown in Figure 1. To investigate the different cross-linking strategies, polymer dispersions with the three different cross-linkers (HEMA, GlyMA, and BPMA) are prepared. The proportions of the cross-linking monomers were varied between 2 and 10 wt%. Table 1 gives a list of the polymers produced.

**Figure 2:**
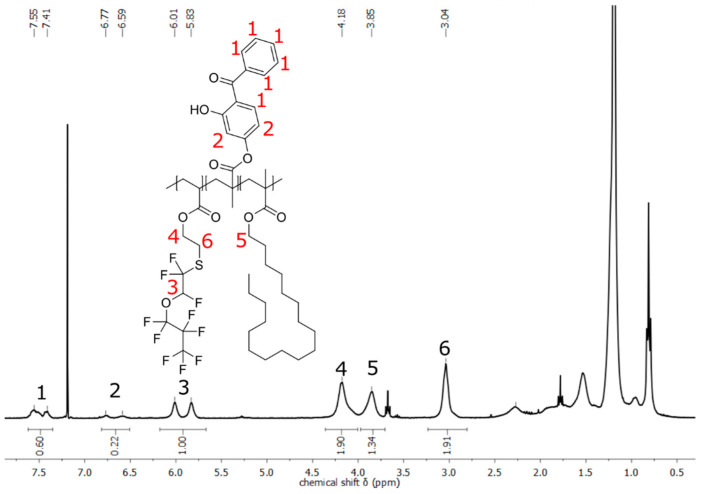
^1^H-NMR spectrum of P(FA-*co*-StMA-*co*-BPMA_5%_) measured in hexafluorobenzene and in CDCl_3_.

While no further preparations are required for the polymerization of HEMA, the polymerization of GlyMA is not trivial. Since the GlyMA can be cross-linked both via acid-catalyzed and base-catalyzed reaction, the pH value must be constant at values of seven to eight during polymerization. This can be accomplished by adding a buffer system of sodium bicarbonate (SBC) and sodium dihydrogen phosphate (SDP) impeding pronounced ring-opening—and therefore—the cross-linking reaction. There are also some challenges when using BPMA as a cross-linking reagent. Since the monomer itself is a solid that is not soluble in water, the monomer must first be dissolved in the mixture of 1,2 propanediol, StMA, and FA prior to dispersing the monomer emulsion with surfactant and water. Despite dissolving the other monomers, no more than 5 wt% of BPMA could be incorporated into the polymer because of solubility issues. Nevertheless, distinct amounts of the monomer BPMA in the polymers are determined by ^1^H-NMR and can be found in Figure 2 and Supplementary Figure S6. The monomer BPMA is a UV cross-linker, so it can also cross-link under the influence of sunlight, which is why the reaction was carried out without exposure to light and the finished polymer was stored in the dark.

The polymer without cross-linking functionalities P(FA-*co*-StMA) was investigated by ^1^H-NMR, SEC, and DSC. A ^1^H-NMR spectrum of the P(FA-*co*-StMA) could be recorded by solving the precipitated polymer in hexafluorobenzene and deuterated chloroform and the respective spectrum is shown in Figure S1. All signals of the P(FA) at 6.45–6.62, 4.14, 3.08, and 2.29 ppm were observed and assigned and are in good agreement with the expectations based on the monomer signals. From the StMA only one signal at 3.83 ppm can be observed, which corresponds to the protons in the vicinity of the ester group. All other proton signals overlap with the backbone signals in the range of 0.5–2.0 ppm. The incorporation ratio of the two monomers FA and StMA was calculated using the signals two and three and lead to 58.6%/41.4% molar ratio, which almost corresponded to the amounts used for the recipe (60%/40%). Molecular weight was measured by size exclusion chromatography (SEC) with PS standards and the corresponding molar mass distribution showed a molar mass of 50.7 kg/mol (M*_n_*) with a polydispersity index, *Đ,* of 1.13 (see Figure S2). The thermal properties of P(FA-*co*-StMA) have been investigated by DSC and revealed a glass transition temperature at −34.9 °C corresponding to P(FA) as well as a melting peak at 38.1 °C caused by P(StMA) (see Figure S3) (*T_g_* P(FA): −36.7 °C [28], T*_m_* P(StMA): 37.8 °C [32,59]).

For characterization of the polymers with cross-linking components, also ^1^H-NMR spectra were recorded. Thus, the cross-linkers HEMA, GlyMA, or BPMA could be observed in the spectra of all polymers of the three different cross-linking strategies, but the proportion of the cross-linking components could not be determined within all spectra. For example, for the polymers P(FA-*co*-StMA-*co*-HEMA) from Table 1, the signals of the protons from HEMA overlap with those of StMA and also partly with some signals of P(FA). Only in the case of the polymer P(FA-*co*-StMA-*co*-HEMA_10%_), the proton of the hydroxy group of HEMA was clearly observed, so the proportion of HEMA could be calculated. By comparing the signals three, four, and five (see spectra Figure S4), a percentage of 8.1% HEMA, 51.1% FA, and 40.8% StMA could be calculated. In the two spectra of polymers two and three, which contain less HEMA, this proton was not clearly detectable and the amount of the integrated cross-linking agent could not be reliably determined. The polymers P(FA-*co*-StMA-*co*-GlyMA) (Nr. 5 to 7 in Figure 2) with GlyMA as the cross-linking agent also showed clear signals in the spectra, but also a clear superposition of the protons of GlyMA with those of StMA. An assignment of the protons can be carried out, as given in Figure S5. For this reason, the proportions of the cross-linker GlyMA cannot be determined accurately here either. However, in the case of polymers eight and nine (P(FA-*co*-StMA-*co*-BPMA)) with BPMA as the cross-linking agent, the proportions could be calculated using the ^1^H-NMR spectra, due to the fact that the aromatic protons of benzophenone do not overlap with those of the two other components. Thus, the signals in the range of 6.5 to 6.8 ppm and in the range of 7.3 to 7.6 ppm can be assigned to the eight protons of BPMA (see Figure 2). The amounts of BPMA in the polymers calculated from ^1^H-NMR spectra are listed in Table 2. The spectrum of polymer eight required for the calculation with 2% BPMA content can be found in Figure S6.

**Figure 3:**
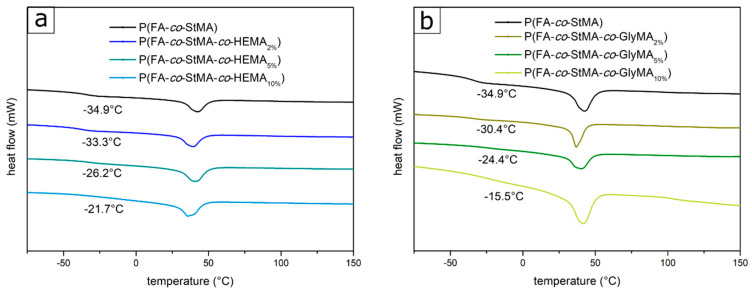
DSC thermogram of (**a**) the polymers copolymerized with HEMA with glass transition temperatures between −34.9 and −21.7 °C and a melting peak at a temperature of 38.1 °C and (**b**) the polymers copolymerized with GlyMA with glass transition temperatures between −34.9 and −15.5 °C and a melting peak at a temperature of 38.1 °C.

The thermal properties of the polymers were determined by DSC measurements. All thermograms of the investigated polymers revealed a melting peak in the range of 37–42 °C [32,59], which was caused by the StMA and a single glass transition temperature. Depending on the amount and type of the cross-linker reagent, the glass transition temperature shifted. The existence of only one *T_g_*, which shifted upon increasing the amount of cross-linker, proved the preparation of a statistical copolymer. Figure 3a shows the thermograms of polymers one to four with HEMA as feasible cross-linking moieties, as shown later. With increasing amounts of HEMA in the polymer, the *T_g_* increased from −34.9 up to −21.7 °C. With the *T_g_* of the homopolymer P(FA) of −36.7 °C (see Figure S3) and the theoretical *T_g_* of P(HEMA) of 80 °C [60] the incorporated fractions of HEMA can be calculated from the respective thermograms. Instead of 2, 5, and 10% HEMA used, actual amounts of 1.8, 5.7, and 8.3% HEMA could be incorporated into the polymer, which is still in good agreement with expectations based on the recipe. The calculated fraction of 8.3% HEMA for the polymer P(FA-*co*-StMA-*co*-HEMA_10%_) was also in good agreement with the ^1^H-NMR spectra from which an amount of 8.1% could be determined. The resulting percentages are also listed in Table 2. The situation is similar with polymers P(FA-*co*-StMA-*co*-GlyMA), whose thermograms are shown in Figure 3b. Here, the glass transition temperature shifted with increasing GlyMA contents from −34.9 °C to −15.5 °C. From the measured *T_g_* and the theoretical *T_g_* of P(GlyMA) of 83 °C [61] it was also possible to estimate GlyMA proportions in the polymers of 3.3, 6.5, and 11.7% instead of 2, 5, and 10%. The calculated amounts are compiled in Table 2.

For polymer P(FA-*co*-StMA-*co*-BPMA) (batch eight and nine), a displacement of the *T_g_* (from −34.9 to −23.1 °C) was also visible and can be concluded from Figure S7. However, no theoretical glass transition temperature of P(BPMA) could be found so far, which means that no amounts could be calculated in this particular case.

**Figure 4:**
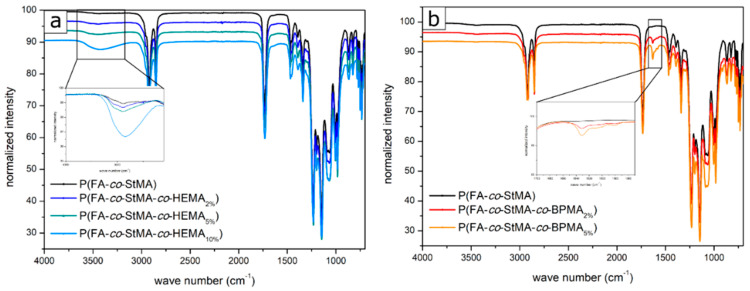
IR spectra of (**a**) the polymers copolymerized with HEMA featuring an increasing signal intensity at 3400 cm^−1^ with increasing proportions of HEMA and (**b**) the polymers copolymerized with BPMA with an increasing signal at 1600 cm^−1^ with increasing proportions of BPMA.

Additionally, the polymers were analyzed by IR spectroscopy. Although this method is not suitable for a quantitative determination of the polymer fractions, considerable qualitative differences could be concluded for the polymers with HEMA as a cross-linkable moiety, as well as for the polymers eight and nine with BPMA as cross-linking moieties. Figure 4a shows the IR spectra of the polymers containing HEMA moieties. In the range from about 3300 to 3500 cm^−1^, a broad band can be observed within all spectra, which could be assigned to the hydroxy-functionalities of P(HEMA). This band is much more pronounced with increasing amounts of HEMA. This observation is comparable to BPMA-containing polymers eight and nine, which showed a small band of aromatic ring vibrations in the wave number range of about 1600 cm^−1^ (see Figure 4b). Again, this absorption was more pronounced when the proportion of BPMA was further increased. The polymers five to seven should have a band at the wavenumber of 910 cm^−1^ [62], which is due to the epoxy functionality of GlyMA. However, this band was not visible as it disappeared in the fingerprint area overlayed by other absorption bands.

In this chapter, the successful preparation of different fluorine-containing polymer dispersions could be demonstrated and clearly revealed the tailorable amount of different cross-linkable moieties as part of the polymer chain. By combining the analytical methods ^1^H-NMR spectroscopy and DSC measurements, the preparation of the respective statistical polymers was proven for all intended strategies.

### 3.2. Application of Polymers on Cellulose Fibers

**Figure 5:**
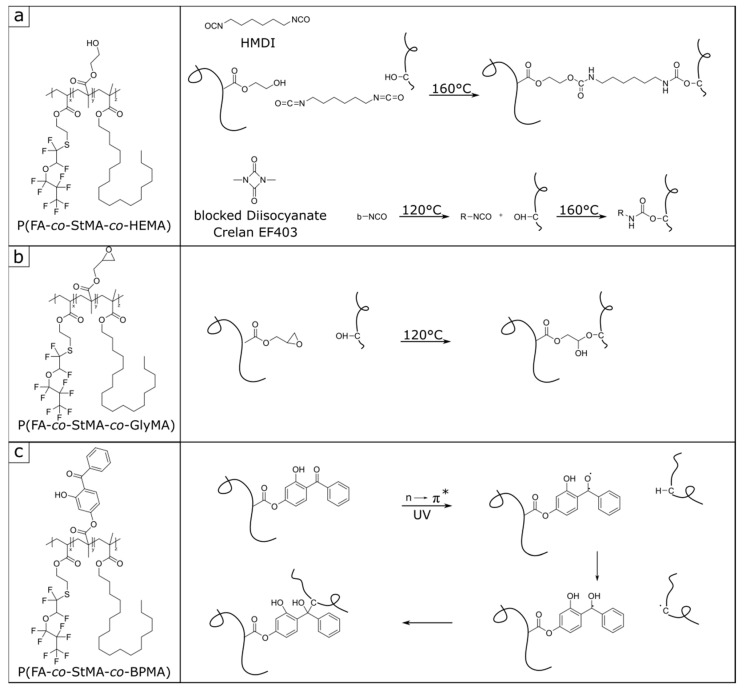
Scheme of the different cross-linking strategies. (**a**) Thermally induced cross-linking reaction with HEMA and two different diisocyanates HMDI and Crelan EF403, as well as the deblocking reaction of Crelan EF403, (**b**) thermally induced cross-linking reaction with GlyMA, and (**c**) UV-induced cross-linking reaction with BPMA [63].

The polymer dispersions were subsequently applied to cellulose filter discs using a size-press laboratory foulard, according to previously described protocols [32]. For this purpose, the different dispersions were diluted to a solid content of 5 wt%, resulting in a comparable polymer content on the cellulose substrates. The dispersions were placed in the laboratory foulard and the paper substrates were coated twice to ensure that they were completely and homogeneously wetted and impregnated. Initially, all paper substrates were first dried at room temperature (19 to 22 °C) and subsequently treated once again in the oven for the respective cross-linking reactions (120 to 160 °C). Figure 5 shows the different cross-linking strategies and the components used.

The paper substrates coated with polymer dispersions two to four featuring HEMA as a cross-linker required the addition of a cross-linkable isocyanate-containing component for the intended thermally induced cross-linking reaction on the cellulose fiber and the bulk material. For this purpose, two different diisocyanates were used, that is, a linear hexamethylene diisocyanate (HMDI), while the other diisocyanate was a blocked cyclic diisocyanate (1,3-dimethyl-1,3-diazetidine-2,4-dione) (Figure 5a), which is known as Crelan EF403. Solutions in dichloromethane (DCM) were prepared from both diisocyanates and the paper substrates coated with the polymer dispersions P(FA-*co*-StMA-*co*-HEMA) were additionally coated with a diisocyanate solution. The two-stage application of the cross-linking components was necessary since the HMDI with its free isocyanate groups would immediately react with water from the aqueous dispersion. The blocked diisocyanate Crelan EF403 is generally not soluble in water and can only be applied from organic solvents. By using DCM, it is assumed that the individual cellulose fibers do not swell as much as in the case of the application from water. It is assumed that the diisocyanate components tend to remain on the surface and in the immediate vicinity of the polymer component. It is worthy to also mention that the binding surface reaction of the diisocyanates with the cellulose fiber is possible in addition to the P(HEMA) cross-linking reaction. The coated substrates (with P(FA-*co*-StMA-*co*-HEMA) + diisocyanate) were first dried at room temperature and treated in the oven at 160 °C overnight to induce the cross-linking (Figure 5a).

To determine the deposited amount of the fluorine-containing polymer on the fibers as a result of the described process, the substrates were weighed before and after the coating step. In order to keep the experimental error due to water sorption as low as possible and to determine the increase in mass due to the polymer coating, all samples were stored and weighed at constant humidity and temperature. The average increase of mass was about 5 to 8%, whereby the fibers coated with the diisocyanates featured higher amounts because of the doubled coating procedure. A table of the found mass increases for all coated substrates can be found in Table S1. An investigation of the amount of polymer absorbed and chemically bound is described in Section 3.4.

Examination of the fibers by IR spectroscopy revealed that for all coated samples, the used polymer was present. For comparison, in Supplementary Figure S8 the IR spectra of uncoated cellulose fibers showing the OH valence vibration band at 3500 cm^−1^ could be observed. In contrast, spectra of the pure polymers and the polymer-coated substrates are additionally given in the Figure, showing the band of cellulose fibers and the carbonyl band at 1700 cm^−1^, which could be assigned to the carbonyl band of the polymer coating.

In addition to IR measurements, coated model fibers made of polyamide (PA) were examined by SEM measurements. Polyamide fibers are used in this case since these fibers all have the same thickness and can therefore be analyzed much better by optical methods than the natural cellulose fibers, which are completely disordered and irregular. Figure S9a shows the corresponding SEM images of uncoated PA fiber and Figure S9b fibers coated with P(FA-*co*-StMA-*co*-HEMA_5%_). While the coated fibers showed a thin film, which was located at the fiber gaps that look rather closed and glued, the uncoated fibers were unglued and lie individually on top of each other. In addition, the diameters of the fibers were measured. The average thickness of the uncoated fiber was determined to be 14.66 µm and the average diameter after coating was 16.02 µm. The fiber diameter increased on average by 1.36 µm due to the coating with the polymer dispersion, which corresponds to a layer thickness of 0.68 µm on the fiber. Also, the fiber thickness coated with the polymers P(FA-*co*-StMA-*co*-GlyMA_5%_) and P(FA-*co*-StMA-*co*-BPMA_5%_) could be measured and the increase in fiber thickness is listed in Supplementary Table S2.

To investigate the performance of coated cellulose fibers, the water and oil repellency of both coated and cross-linked substrates was evaluated. For water repellency, a static contact angle was measured with a sessile water drop with a volume of 4 µL. For this purpose, a drop of water was deposited on the substrate and the shape of the spherical drop was measured. If the fiber was too hydrophilic, the droplet directly penetrated into the substrate. The more hydrophobic the substrates, the longer the droplet remained on the surface or even impeded wetting of the fiber substrate. The water contact angles for all substrates were collected and compiled (Table 3). The oil repellency of the fibers was measured using the American Association of Textile Chemists and Colorists (AATCC) Test method 18 (hydrocarbon test), in which a number of different test liquids with different hydrocarbon chains and surface tensions were applied to the fibers and the wetting of the fibers by the oil was evaluated. For each oil repellency, a grade could be assigned in order to indicate, which oil was not capable of wetting the fibers. In brief, a high number corresponded to a good oil repellency (Table 3). A table with the test oils, the corresponding grading, and the surface tensions of the liquids can be found in Table S3.

It can be concluded from the collected data in Table 3 that the untreated paper substrates are neither water nor oil repellent. The fibers coated with P(FA-*co*-StMA) already featured a good water repellency with a water contact angle (WCA) of 139°. A value of grade four was achieved in the test for the oil repellency. For all polymers containing cross-linking components, it can be observed that the contact angle was usually similar to the polymer P(FA-*co*-StMA) or sometimes even higher, despite the fact that the copolymerization of HEMA, GlyMA, or BPMA introduced additional polar functionalities. In particular, thermally induced cross-linking with HEMA and HMDI, as well as the thermal cross-linking reaction with GlyMA, still revealed values of around 146° for the WCAs. The oil repellency was also surprisingly high with GlyMA as the cross-linking reagent leading to a value of six for cross-linking reagents contents of 5 to 10%, which turned out to be much higher than without cross-linking reagents. Only in the case for the UV-induced cross-linking strategy with BPMA as a cross-linker caused only little improvement leading to similar values for water and oil repellency as with the non-cross-linked polymers.

Based on the previous analysis, such as the mass increase of the fiber, the IR spectra and SEM model analysis as well as the good repellent performance both for water and for oils, it can be proven that the polymers were successfully applied to the fiber substrates. The slightly increased contact angles with increasing degree of cross-linking give a hint that the intended cross-linking reaction to the fibers was successful. In order to also prove the cross-linking reaction with the substrate, additional investigations will be demonstrated in the ensuing section.

### 3.3. Investigation and Quantification of Cross-Linking Reactions

**Figure 6:**
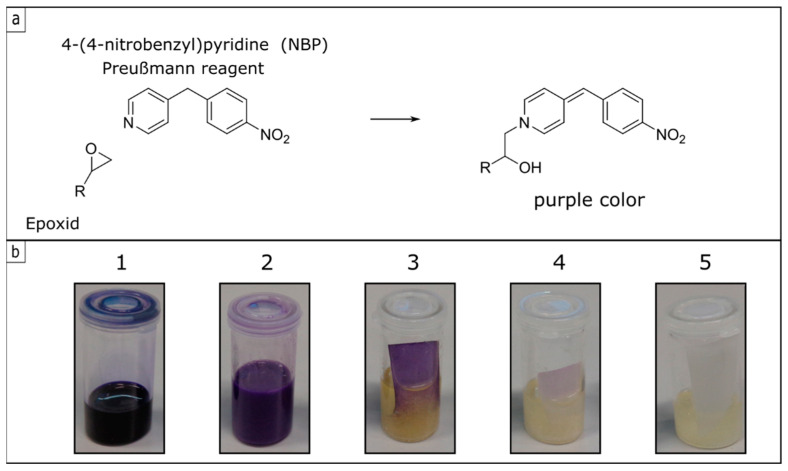
(**a**) Preußmann reagent 4-(4-nitrobenzyl)pyridine (NBP) and the reaction with an epoxide to the violet color, (**b**) NBS stock solution with various samples in them (1 = pure monomer GlyMA, 2 = P(FA-*co*-StMA-*co*-GlyMA_10%_), 3 = P(FA-*co*-StMA-*co*-GlyMA_10%_) on cellulose fibers prior to the cross-linking reaction, 4 = P(FA-*co*-StMA-*co*-GlyMA_10_) on cellulose fibers after the cross-linking reaction, and 5 = pristine cellulose fiber substrate).

In order to underpin the successful cross-linking strategy of the fiber substrates, an optical experiment was selected in the case of the GlyMA-modified substrates. This experiment involves the reagent 4-(4-nitrobenzyl)pyridine (NBP)—also referred to as the Preußmann reagent—which can be used to achieve an efficient ring-opening reaction of epoxy groups. The reaction with epoxides will be made visible because of an accompanied intense violet coloration upon the ring-opening reaction of the dye [64]. Figure 6 shows the Preußmann reagent reaction with an epoxy group along with photographs for coloration in solution and on the coated cellulose substrates. By using NBP, a qualitative statement could be made whether the epoxy functionalities were present or whether these epoxy groups had already reacted. When epoxides were added to an NBP solution (50 mg NBS in 5 mL isopropanol and five drops of ammonia), the ring-opening reaction takes place resulting in an intense purple coloration based on the amount of present epoxides. If there were no residual epoxy functionalities within the sample or cellulose substrate surface, the coloration would remain colorless to slightly yellowish (Figure 6b).

Figure 6b shows photographs of the samples treated with the NBS stock solution, whereupon different color changes could be observed. Sample 1 contained the pure monomer GlyMA with the active epoxy moieties, so that a positive Preußmann test and an intense coloration could be achieved. Sample 2 contained the polymer dispersion P(FA-*co*-StMA-*co*-GlyMA_10%_). As a finding, the epoxy functionalities were active after the starved-feed emulsion polymerization, which was also proven by the intense violet coloration. The cellulose substrate treated with the respective polymer dispersion was also added to the stock solution of NBP (sample three) and after 30 min a first coloration of the substrate was detected. After one hour, a strong purple color appeared. In this case, the polymer was present on the fiber substrate, but obviously, the epoxide functionalities were still active and present at this stage. Finally, sample four shows the polymer-coated cellulose substrate (with P(FA-*co*-StMA-*co*-GlyMA_10%_)) additionally treated and cross-linked in an oven at 120 °C overnight. Testing the thermally treated substrates with the NBP solution again, only a slight violet coloration could be detected after a few hours. This observation indicated that most of the epoxy functionalities already reacted both with the cellulose substrates and the polymer coating after the thermal treatment. Sample five shows a reference substrate without coating. Here it can be observed that a completely negative Preußmann test resulted in a slightly yellowish coloration under the same conditions.

**Figure 7:**
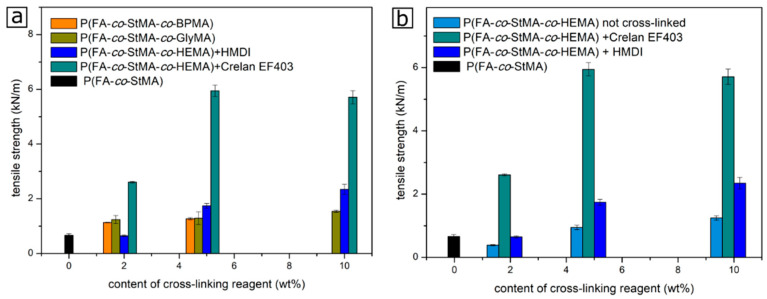
(**a**) Obtained values for the tensile strength of the non-cross-linked and cross-linked cellulose fiber substrates. (**b**) Comparison of tensile strength values for cellulose fibers coated with P(FA-*co*-STMA-*co*-HEMA) and after thermally induced cross-linking reaction with the different diisocyanates.

For an evaluation of the cross-linking strategies and the assessment of the efficiency of the performed cross-linking reaction, tensile elongation tests were carried out for the coated cellulose fiber substrates. For this purpose, the cellulose substrates were coated with different polymer dispersions, as described above, followed by subsequent cross-linking protocol, which was either thermally induced or the application of UV radiation. From the cellulose fibers, strips with a width of 1.5 cm and a length of at least 12 cm were cut and the tensile strength, as well as the elongation profile, were recorded. It is considered that the cross-linking of the polymer takes place on top of the fiber, and therefore, new covalent bonds were formed resulting in an increased tensile strength dependent on the amount of used cross-linking reagents. Figure 7 compares the tensile strength of the different cross-linked substrates.

The tensile strength was measured for at least five samples and an average value and error bars were calculated. The polymer P(FA-*co*-StMA) is given in black. Since this polymer is not capable of cross-linking with the cellulose fiber, a rather low tensile strength value of approximately 0.6 kN/m was found. Comparing the different cross-linking strategies, it can be concluded that the highest tensile strength was achieved with P(FA-*co*-STMA-*co*-HEMA) and cross-linked with the blocked diisocyanate Crelan EF403 at a cross-linking amount of 5 to 10%. For both cross-linking amounts, the obtained tensile strength was found to be 6 kN/m. Moreover, a lower cross-linking content of 2% revealed a significantly increased tensile strength compared to the non-cross-linked samples. Increased tensile strength values could also be determined when cross-linking was carried out with GlyMA and BPMA. Figure 7b summarizes the tensile strength of the fiber coated with the polymer P(FA-*co*-StMA-*co*-HEMA) and with different diisocyanates and additionally without any cross-linking reagent. It can be concluded from these results that the polymer P(FA-*co*-STMA-*co*-HEMA_2%_) without any cross-linking component even shows a lower tensile strength than P(FA-*co*-StMA). As a result, from Figure 7a, the cross-linking with Crelan EF403 provided the highest tensile strength values and thus can be considered as the most efficient cross-linking protocol. Although both cross-linking components were diisocyanates, the results significantly differed. The increased tensile strength with Crelan EF403 could be due to its rigid structure, while HMDI still featured a more flexible character because of the long hexyl-chain, or by an increased reactivity and, therefore, more effective cross-linking. It is assumed that the blocked Crelan EF403 represents a short and less flexible structure that can more strongly fix the polymer to the fibers and their surfaces. Based on these results HMDI could be expected to have a higher elasticity than Crelan EF403. A comparison of the polymers P(FA-*co*-StMA-*co*-BPMA) cross-linked by UV irradiation and the respective non-treated blank sample is shown in Figure S10. Again, the polymer that was not cross-linked was comparable to the polymer P(FA-*co*-StMA). Hence, only a cross-linking reaction upon irradiation in the UV cube could increase the tensile strength values.

**Figure 8:**
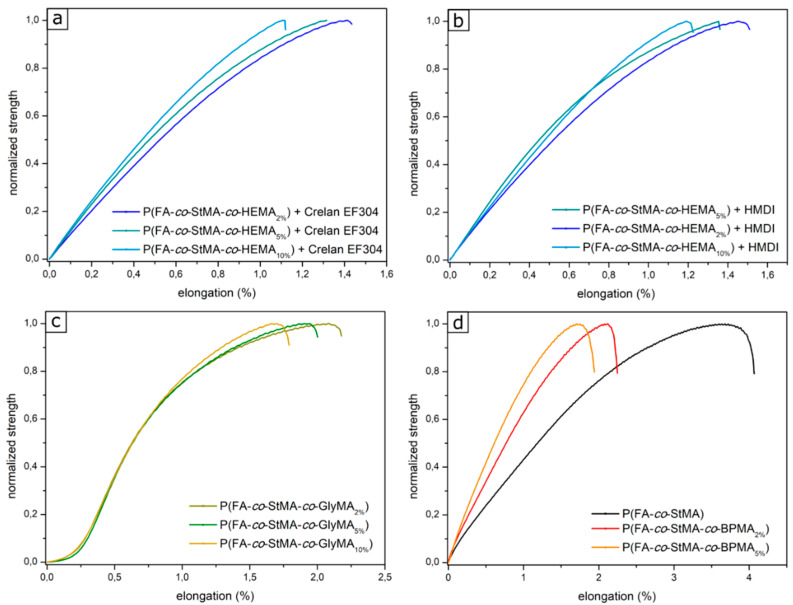
Elasticity plot of the cross-linked cellulose fibers coated with (**a**) P(FA-*co*-StMA-*co*-HEMA) cross-linked with Crelan EF403, (**b**) P(FA-*co*-StMA-*co*-HEMA) cross-linked with HMDI, (**c**) P(FA-*co*-StMA-*co*-GlyMA) thermal-induced cross-linked, and (**d**) P(FA-*co*-StMA-*co*-BPMA) UV-induced cross-linked, as well as the non-cross-linked fibers.

In addition, Figure 8 shows the elongation of cellulose substrates. Five measurements per sample were performed and the measurement with the best representation is shown. It can be expected that an increased cross-linking and thus an increased number of covalent bonds between the polymer and the individual fibers will reduce the elongation of the samples. As a result, from Figure 8a,b, in the polymer P(FA-*co*-StMA-*co*-HEMA) with the diisocyanate Crelan EF403 and with HMDI this behavior could be concluded. A significant increase of elongation was observed for the non-cross-linked sample, whereas the comparison of the two diisocyanates HMDI and Crelan EF403 revealed the expected difference of elongation.

In Figure 8c, the result of cross-linking with GlyMA is shown and the most rigid cellulose fiber substrates could be obtained with a higher percentage of cross-linking amount in the polymer. Compared to this, a lower amount of GlyMA revealed the most elastic behavior for the coated substrates. Similar trends were observed for cross-linking with BPMA (see Figure 8d). Supplementary Figure S11c shows all polymers with a cross-linking content of 5% for the investigated strategies. It is obvious that the cross-linking of GlyMA and BPMA yielded a similar effect, but these samples were much more elastic than the cross-linking reaction with HEMA.

For practical application, it is also of interest that the cross-linking features enable a positive effect on the wash-resistance coating, which will be described in the following section.

### 3.4. Chemical Integrity under Various Forces

**Figure 9:**
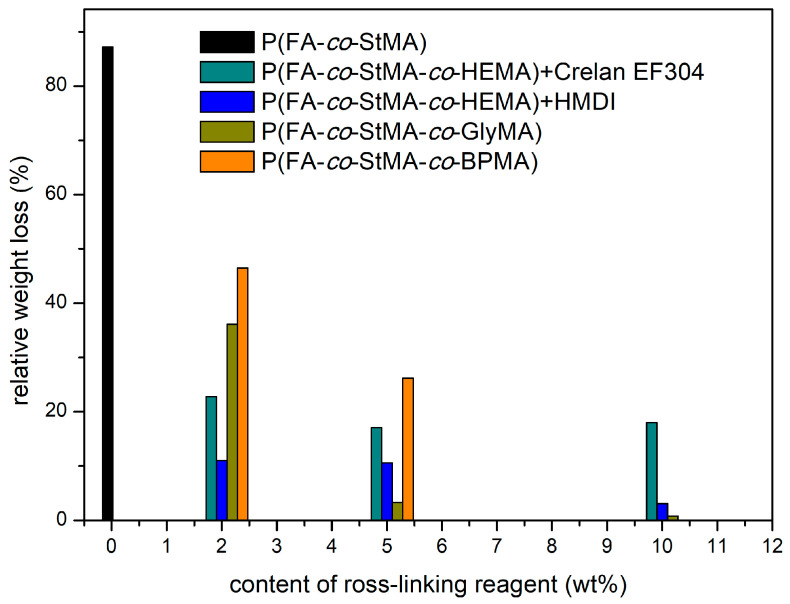
Weight loss of polymer-coated fibers by extraction in the Soxhlet apparatus, relative to the initial coated cellulose substrate weight.

In order to investigate the desired effect on wash-resistance, the fluorine-containing polymer coating and cross-linked cellulose fiber substrates were subjected to an extraction process that repeatedly simulated the washing of the substrates. For this purpose, a Soxhlet apparatus was equipped with the corresponding cellulose substrates and extraction was carried out for 24 h. Since the polymer was not soluble in water, the substrates were subjected to a THF a solvent, which turned out to be a good solvent for the polymers. The quantitative analysis of the extraction could be followed by the mass loss. For this purpose, all obtained masses are listed in Supplementary Table S4 and compiled in the bar chart in Figure 9.

Supplementary Table S4 lists the masses of the uncoated papers, the masses of coated papers, and the masses of extracted papers, from which the percentual loss of polymer weight by extraction or by the repeatedly simulated washing process could be calculated. It can be concluded from these results that the non-cross-linked polymer P(FA-*co*-StMA) (black) lost 87% of its mass upon the extraction procedure, featuring a final residue of 13% polymer on the fibers. For all three cross-linking strategies it can be observed that the loss of mass was significantly lower than for the non-cross-linked polymer, therefore efficient cross-linking of the polymers could be proven. Moreover, it can also be concluded that the weight loss of any given polymer decreased as the degree of cross-linking increased, which was in line with expectations. While UV cross-linking with BPMA still indicated a relatively large reduction with respect to polymer mass (–46% for P(FA-*co*-StMA-*co*-BPMA_2%_) and −26% at P(FA-*co*-StMA-*co*-BPMA_5%_)), the mass loss of thermally cross-linked polymers could be reduced to 0.3% (via cross-linking reaction with P(FA-*co*-StMA-*co*-GlyMA_10%_)). It is assumed, that despite the fact that the paper samples were irradiated with 1000 W UV light from each side for 5 min, the cross-linking reaction could not occur in the interior of the cellulose fiber substrate. Therefore, only cross-linking moieties near the surface of the entire substrate could be addressed upon UV irradiation. In summary and in comparison, the thermally induced cross-linking strategy with HEMA and the diisocyanates lead to a significantly reduced weight loss, so that a more efficient cross-linking was proven.

**Figure 10:**
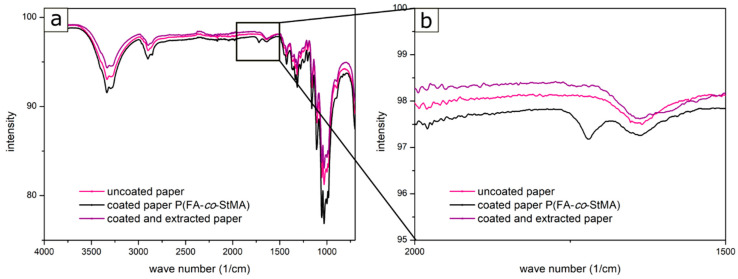
(**a**) IR spectra of the uncoated cellulose fiber substrate (pink), the cellulose fiber coated with P(FA-*co*-StMA) (black), and the cellulose fiber coated and extracted (violet) featuring a signal at 1700 cm^−1^ for the carbonyl-vibes and (**b**) magnification of the area at 1700 cm^−1^.

In addition to the gravimetric determination, IR spectra of the coated and extracted cellulose fibers were recorded. The cellulose paper substrate without coating, coated substrate, and a coated, cross-linked, and extracted substrate were directly compared. Figure 10 shows the spectra for the non-cross-linked polymer P(FA-*co*-StMA). Due to the weight loss, it was already clear that nearly all of the polymer had been washed off the fibers. This could also be detected by collecting the IR spectra. Especially, the carbonyl band in the range of about 1700 cm^−1^ indicated the presence of the polymer on the fibers and this signal almost completely vanished after extraction. The range around the wave number 1700 cm^−1^ was additionally magnified in Figure 10b to be clearer. While the coated paper featured a corresponding absorption band (black spectra), the coated and extracted paper was almost identical to the uncoated substrate, indicating that the polymer was almost completely removed by the washing process.

The IR spectra of the polymers P(FA-*co*-StMA-*co*-HEMA_10%_) + Crelan EF403, P(FA-*co*-StMA-*co*-HEMA_10%_) + HMDI, P(FA-*co*-StMA-*co*-GlyMA_10%_) and P(FA-*co*-StMA-*co*-BPMA_5%_), and the magnifications of the carbonyl band in the range of 1700 cm^−1^ are given in SI-16. The thermally induced cross-linking with HEMA- or GlyMA-containing copolymers were identical to the non-extracted samples. The carbonyl band was still present, and the polymer could not be removed upon the extraction step. In Figure S12d), in the samples that were UV cross-linked with BPMA, the carbonyl moiety was slightly reduced upon extraction of the substrates. This again indicated that the polymer was partly removed from the fiber surface because of the insufficient cross-linking reaction. This result also corresponded to the weight loss from Figure 9. Since the weight loss and the IR spectra demonstrated that the cross-linked polymers could not be removed upon the simulated washing process, the performance of the substrates with respect to wetting after the extraction will be investigated in the following. Again, the water repellency by static contact angle and the oil repellency according to the hydrocarbon test were performed and the results were compared with the water and oil repellency values prior to extraction (Table 4).

The paper substrates with the non-cross-linked polymer neither revealed a water- nor an oil-repellency after extraction, so it can be concluded that cross-linking turned out to be necessary to lead to improved repelling performances. The water repellency of all other polymers cross-linked on the fiber remained similar to that before extraction. Although the values for water repellency slightly differed, the changes were in the range of the experimental error for this method. Even the UV cross-linked substrates, which revealed a weight loss of almost 50% for the polymer during extraction, a high water-repellency was still achieved. This indicated that a good protection performance could still be reached even with significantly less polymer on the cellulose fibers. A comparison of the oil-repellency of the substrates prior to and after the extraction revealed slight differences. The oil repellency was reduced after extraction for almost all cross-linking types; only thermal cross-linking with 10% HEMA and HMDI as diisocyanate still featured the same oil repellency with a grade of six.

**Figure 11:**
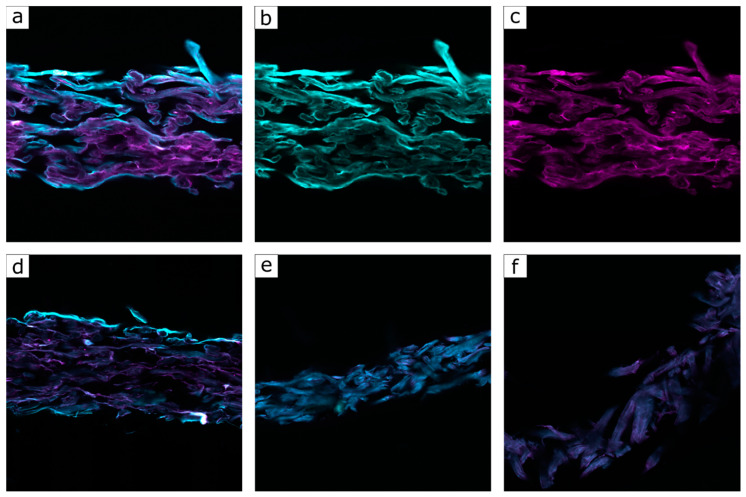
Confocal laser scanning microscopy (CLSM) images of cellulose fibers. (**a**–**c**) coated with P(FA-*co*-StMA-*co*-HEMA-*co*-RhBMA); (**a**) calcofluor channel and RhBMA channel, shows the fiber and polymer, (**b**) calcofluor channel shows the fibers, (**c**) RhBMA channel shows the polymer. (**d**–**f**) Coated with P(FA-*co*-StMA-*co*-HEMA-*co*-RhBMA), (**d**) untreated fiber with the calcofluor channel and the RhBMA channel, (**e**) non-cross-linked coating, but extracted fiber, (**f**) cross-linked and extracted fiber.

Since cross-linking protocols with the polymer P(FA-*co*-StMA-*co*-HEMA_10%_) with HMDI was the most effective method with respect to its water- and oil-repellency, as well as the weight loss after extraction, finally, we would like to address the question of whether only the exterior of the fiber or the interior or even the entire fiber substrate was coated with the polymer. For this purpose, the polymer on the fibers was visualized by means of confocal laser scanning microscopy (CLSM). To follow this question, a fluorescent monomer, that is, Rhodamine B meth acrylamide (RhBMA) was synthesized according to our protocols [53,54] (Figure S14) and copolymerized with FA, StMA, and HEMA as described in Section 2.3 (54.75% FA, 34.75% StMA, 10% HEMA, and 0.5% RhBMA). Since there was only 0.5 wt% of the RhBMA content in the polymer, this monomer was not expected to change the wetting properties of the final polymer. As a first hint to the successful synthesis, the polymer dispersion featured an intense pink coloration due to the presence of the dye. The polymer dispersion was applied to the fibers as described in Section 2.4. The coated paper fibers were soaked in a calcofluor white solution, which was used to dye the cellulose fibers for the CLSM [65,66]. Calcofluor white is a fluorescent blue dye based on a diamino stilbene, which is particularly suitable for binding to cellulose, polysaccharides, or chitin structures. Due to the different wavelengths used to excite the two fluorescent dyes, the fibers and the polymer could be individually visualized during the CLSM. For the measurement, the colored samples were embedded in an epoxy resin and cut into 80 µm thin samples. In this way, not only the surface but also the interior of the fiber could be examined. Figure 11 shows the images taken from these investigations.

Figure 11a–c illustrates the cellulose fibers that were only coated and dyed. Figure 11a shows an overlay of the fluorescence channels of both fluorophores, calcofluor white and RhBMA. The fibers are displayed in turquoise, while the polymer is shown in pink. For imaging, two lasers exciting the fluorophores at 405 and 552 nm were used and the emission was measured between 420 and 470, and 575 and 630 nm for the two dyes calcofluor white and RhBMA, respectively. Accordingly, Figure 11b shows only the individual fibers with calcofluor white-colored fibers and Figure 11c the pink copolymer P(FA-*co*-StMA-*co*-HEMA-*co*-RhBMA). The main feature within these photographs that could be observed was the uniform coating of the fibers with the respective RhBMA-containing copolymer.

To further investigate the chemical integrity of the polymer, a paper substrate was coated with the polymer and colored with calcofluor white. A sample of the non-cross-linked substrate and a sample of the cross-linked polymer substrate was extracted for 24 h in order to determine the degree to which the polymer was washed off the fiber. A non-cross-linked and not-extracted sample is shown in Figure 11d. The fibers are colored in turquoise and the polymer is evenly distributed in pink. Figure 11e illustrates the non-cross-linked sample after extraction and a significant decrease of polymer could be observed. Only isolated slight traces of the pink dye could be observed. Overall, the fibers appeared somewhat shorter and more frayed, which suggested that the extraction over 24 h with THF caused some damage to the cellulose fibers. Figure 11f depicts the cross-linked polymer after extraction on the fiber. The fibers seem to be shortened and frayed, however, the pink color of the polymer was still visible. This optical indication of the weight loss after extraction as well as the performance in terms of water and oil repellency turned out to be in line with the previously described experiments (tensile strength tests and others). Therefore, it can be concluded that an efficient thermal cross-linking of the polymer on the cellulose fiber was achieved, which was capable of resisting an intense organic solvent treatment (THF).

In a comparison of the three strategies, a feasible cross-linking method could be demonstrated for all polymers, which resulted in a higher performance and resistance of the polymer on the fiber. Moreover, all investigations revealed that thermal cross-linking is preferable to UV cross-linking, as this UV cross-linking was less effective. In this case, even better cross-linking results could be achieved by varying the irradiation time and power and adding a bifunctional monomer. In a comparison of the thermal cross-linking strategies, both P(FA-*co*-StMA-*co*-HEMA) as well as P(FA-*co*-StMA-*co*-GlyMA) performed with similar success. Both strategies demonstrated nearly complete cross-linking at higher cross-linking degrees of approximately 5 to 10%. Tensile elongation tests, as well as water repellency, indicated that cross-linking with P(FA-*co*-StMA-*co*-HEMA) is more successful. The disadvantage of this method is that a two-step coating of the fiber is required.

## 4. Conclusions

In conclusion, an efficient protocol for the preparation of a fluorine-containing polymer for application on textiles with water- and oil-repellent properties was described. Three different cross-linking strategies were presented and investigated in detail. To gain insights into the efficiency of the cross-linking reaction, tensile-elongation experiments were carried out, which demonstrated an increased tensile strength and a reduced elongation of the cross-linked fibers in comparison to the non-crosslinked fibers for all investigated strategies. To investigate the macroscopic effect of the chemical cross-linking reactions, a washing process was mimicked and the cellulose substrates were characterized again. For optical analysis, a fluorescent dye was incorporated into the polymer and analyzed on the fiber by using confocal microscopy. In conclusion, no or no significant loss of the cross-linked polymer during extraction was found and a uniform repellency to oil and water could be achieved. The latter indicated a high chemical integrity, even when good solvents for the polymers were applied to the coated substrates. All three cross-linking strategies were shown to be highly effective and therefore demonstrated their durable water- and oil-repellency feature of the fluoropolymer coating on the fibers. In conclusion, the cross-linking strategy of the polymer P(FA-*co*-StMA-*co*-HEMA) with diisocyanates provided the best performance in terms of functional surfaces.

## Figures and Tables

**Table 1:** List of the polymers synthesized with FA, StMA, and the cross-linking reagent X (HEMA, GlyMA, BPMA) with all masses of monomers used.

	Polymer	m_(FA)_ (g)	m_(StMA)_ (g)	m_(X)_ (g)
1	P(FA-*co*-StMA)	20.56	13.54	0
2	P(FA-*co*-StMA-*co*-HEMA_2%_)	20.22	13.20	0.68
3	P(FA-*co*-StMA-*co*-HEMA_5%_)	19.70	12.69	1.71
4	P(FA-*co*-StMA-*co*-HEMA_10%_)	18.85	11.85	3.41
5	P(FA-*co*-StMA-*co*-GlyMA_2%_)	20.22	13.20	0.68
6	P(FA-*co*-StMA-*co*-GlyMA_5%_)	19.70	12.69	1.71
7	P(FA-*co*-StMA-*co*-GlyMA_10%_)	18.85	11.85	3.41
8	P(FA-*co*-StMA-*co-*BPMA_2%_)	20.22	13.20	0.68
9	P(FA-*co*-StMA-*co*-BPMA_5%_)	19.70	12.69	1.71

**Table 2:** List of the proportions of monomers used, as well as the polymerized proportions calculated by ^1^H-NMR and DSC.

	Polymer	Assigned Fractions FA/StMA/X (%)	Fractions Calculated from 1H-NMR FA/StMA/X (%)	Fractions Calculated from DSC Thermograms FA/StMA/X (%)
2	P(FA-*co*-StMA-*co*-HEMA_2%_)	59.0/39.0/2.0	/	59.1/39.1/1.8
3	P(FA-*co*-StMA-*co*-HEMA_5%_)	57.5/37.5/5.0	/	57.2/37.1/5.7
4	P(FA-*co*-StMA-*co*-HEMA_10%_)	55.0/35.0/10.0	51.1/40.8/8.1	55.9/35.8/8.3
5	P(FA-*co*-StMA-*co*-GlyMA_2%_)	59.0/39.0/2.0	/	58.4/38.3/3.3
6	P(FA-*co*-StMA-*co*-GlyMA_5%_)	57.5/37.5/5.0	/	56.8/37.7/6.5
7	P(FA-*co*-StMA-*co*-GlyMA_10%_)	55.0/35.0/10.0	/	54.2/34.1/11.7
8	P(FA-*co*-StMA-*co-*BPMA_2%_)	59.0/39.0/2.0	62.2/36.2/1.6	/
9	P(FA-*co*-StMA-*co*-BPMA_5%_)	57.5/37.5/5.0	55.3/38.8/5.9	/

**Table 3:** Contact angles of water for the uncoated and coated cellulose fiber substrates, and the oil repellency of the fibers tested with the hydrocarbon test (4 = *n*-tetradecane, 5 = *n*-dodecane, 6 = *n*-decane).

Fiber Coated with Polymer	Contact Angle	Oil Repellency
Uncoated fiber	0°	0
P(FA-*co*-StMA)	139 ± 3°	4
P(FA-*co*-StMA-*co*-HEMA_2%_) + HMDI	138 ± 2°	4
P(FA-*co*-StMA-*co*-HEMA_5%_) + HMDI	140 ± 3°	5
P(FA-*co*-StMA-*co*-HEMA_10%_) + HMDI	142 ± 4°	6
P(FA-*co*-StMA-*co*-HEMA_2%_) + Crelan EF403	134 ± 3°	4
P(FA-*co*-StMA-*co*-HEMA_5%_) + Crelan EF403	135 ± 1°	6
P(FA-*co*-StMA-*co*-HEMA_10%_) + Crelan EF403	140 ± 3°	6
P(FA-*co*-StMA-*co*-GlyMA_2%_)	141 ± 4°	6
P(FA-*co*-StMA-*co*-GlyMA_5%_)	145 ± 6°	6
P(FA-*co*-StMA-*co*-GlyMA_10%_)	146 ± 4°	6
P(FA-*co*-StMA-*co-*BPMA_2%_)	138 ± 1°	4
P(FA-*co*-StMA-*co*-BPMA_5%_)	140 ± 3°	5

**Table 4:** List of the contact angles against water for the non-extracted and extracted cellulose fibers, as well as the oil repellency of the fibers tested with the hydrocarbon test (4 = *n*-tetradecane, 5 = *n*-dodecane, 6 = *n*-decane) before and after extraction for 24 h with THF.

Fiber Coated with Polymer	Contact Angle before Extraction	Oil Repellency before Extraction	Contact Angle after Extraction	Oil Repellency after Extraction
Uncoated fiber	0	0	0	0
P(FA-*co*-StMA)	139 ± 3	4	0	0
P(FA-*co*-StMA-*co*-HEMA_2%_) + HMDI	138 ± 2	4	136 ± 2	4
P(FA-*co*-StMA-*co*-HEMA_5%_) + HMDI	140 ± 3	5.5	139 ± 4	5
P(FA-*co*-StMA-*co*-HEMA_10%_) + HMDI	142 ± 4	6	138 ± 1	6
P(FA-*co*-StMA-*co*-HEMA_2%_)+Crelan EF403	134 ± 3	4	135 ± 4	4
P(FA-*co*-StMA-*co*-HEMA_5%_)+Crelan EF403	135 ± 1	6	135 ± 2	5.5
P(FA-*co*-StMA-*co*-HEMA_10%_)+Crelan EF403	140 ± 3	6	137 ± 1	5.5
P(FA-*co*-StMA-*co*-GlyMA_2%_)	141 ± 4	6	140 ± 6	5.5
P(FA-*co*-StMA-*co*-GlyMA_5%_)	145 ± 6	6	143 ± 4	5.5
P(FA-*co*-StMA-*co*-GlyMA_10%_)	146 ± 4	6	144 ± 3	5.5
P(FA-*co*-StMA-*co-*BPMA_2%_)	138 ± 1	4	133 ± 3	4
P(FA-*co*-StMA-*co*-BPMA_5%_)	140 ± 3	5	137 ± 2	4.5

## Data Availability

The data presented in this study are available on request from the corresponding author.

## Supplementary Materials

The following are available online at https://www.mdpi.com/2073-4360/13/5/723/s1. Figure S1: ^1^H-NMR of P(FA-*co*-StMA), Figure S2: SEC measurement of P(FA-*co*-StMA), Figure S3: DSC thermogram of P(FA), P(StMA), and P(FA-*co*-StMA), Figure S4: ^1^H-NMR of P(FA-*co*-StMA-co-HEMA_10%_), Figure S5: ^1^H-NMR of P(FA-*co*-StMA-co-GlyMA_10%_), Figure S6: ^1^H-NMR of P(FA-*co*-StMA-co-BPMA_2%_), Figure S7: DSC thermogram of P(FA-*co*-StMA-co-BPMA), Table S1: List of mass increase of cellulose substrates, Figure S8: IR analysis of P(FA-*co*-StMA-*co*-HEMA_5%_), P(FA-*co*-StMA-*co*-GlyMA_5%_), and P(FA-*co*-StMA-*co*-BPMA_5%_), Figure S9: SEM images of PA fibers coated with P(FA-*co*-StMA-*co*-HEMA_5%_), P(FA-*co*-StMA-*co*-GlyMA_5%_), and P(FA-*co*-StMA-*co*-BPMA_5%_), Table S2: List of average increase of PA-fiber diameters, Table S3: Test liquids, oil repellency grades and surface tension of the hydrocarbon test, Figure S10: Tensile strength of P(FA-*co*-StMA-*co*-BPMA_5%_), Figure S11: Comparison of the elongation of P(FA-*co*-StMA-*co*-HEMA_5%_), P(FA-*co*-StMA-*co*-GlyMA_5%_) and P(FA-*co*-StMA-*co*-BPMA_5%_), Table S12: List of weight loss of polymers, Figure S13: IR spectra of coated and extracted papers with P(FA-*co*-StMA-*co*-HEMA_10%_), P(FA-*co*-StMA-*co*-GlyMA_10%_), and P(FA-*co*-StMA-*co*-BPMA_5%_), Figure S14: Synthesis of Rohdamin B meth arylamide (RhBMA).
