# Supplementary material for: Cross-Linking Strategies for Fluorine-Containing Polymer Coatings for Durable Resistant Water- and Oil-Repellency
Source: Polymers (Basel). 2021 Feb 27;13(5):723. doi: 10.3390/polym13050723 (PMC7956606; doi:10.3390/polym13050723)
Supplement: Supplementary file 1 [file polymers-13-00723-s001.pdf]

Supporting Information

Cross-Linking Strategies for Fluorine-Containing Polymer Coatings for Durable Resistant Water and Oil-Repellency

Julia Kredel [1,2], Deborah Schmitt [1], Jan-Lukas Schäfer [2], Markus Biesalski [2] and Markus Gallei [1,*]

[1] Chair in Polymer Chemistry, Universität des Saarlandes, Campus Saarbrücken, 66123 Saarbrücken, Germany, markus.gallei@uni-saarland.de (M.G.)

[2] Ernst-Berl Institute of Technical and Macromolecular Chemistry, Technische Universität Darmstadt, Alarich-Weiss-Straße 4, 64287 Darmstadt, Germany

* Correspondence: markus.gallei@uni-saarland.de

Corresponding Author

*Markus Gallei - markus.gallei@uni-saarland.de

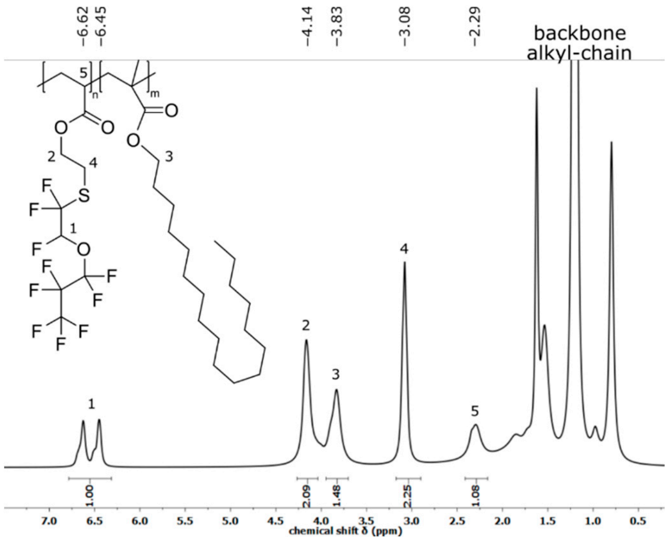

Figure S1. ^{1}H-NMR of P(FA-*co*-StMA) in hexafluoro benzene and $CDCl_3$. Incorporated amount of FA/StMA is 58.6/41.4.

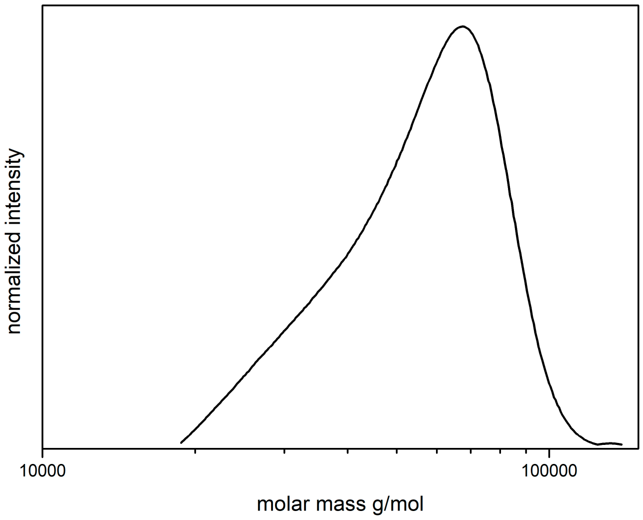

Figure S2. SEC measurement of P(FA-*co*-StMA). Mn: 50.7 kg/mol, dispersity 1.13.

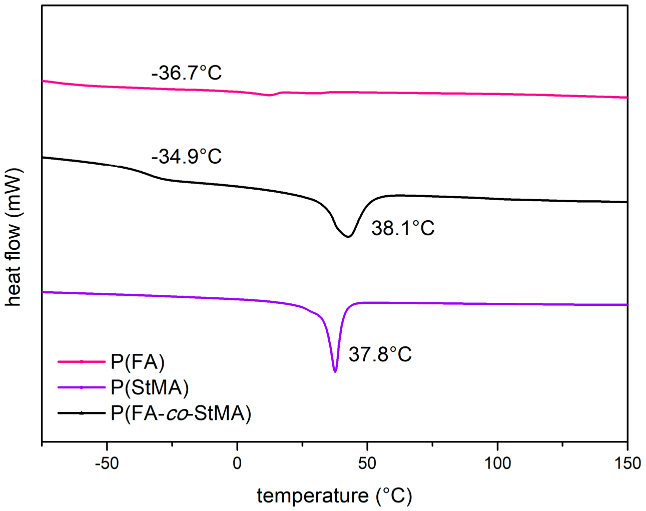

Figure S3. DSC measurements of P(FA) (pink), P(StMA) (purple), and the copolymer P(FA-*co*-StMA) (black).

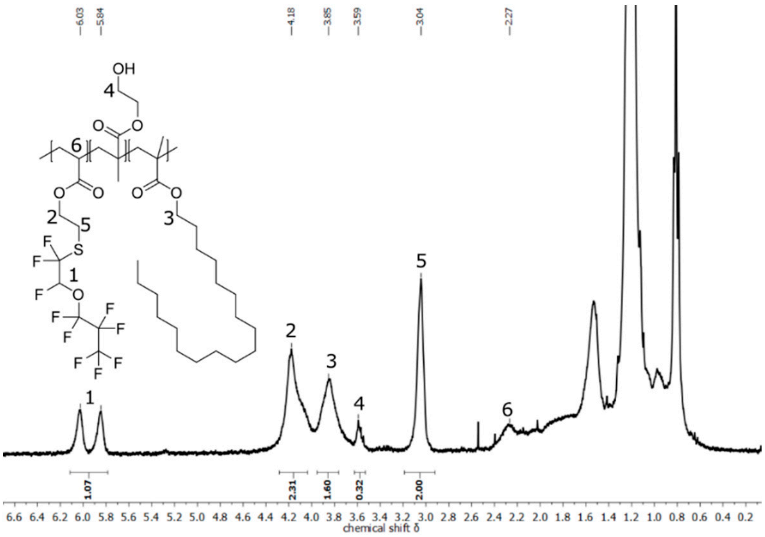

Figure S4. ^{1}H-NMR of P(FA-*co*-StMA-*co*-HEMA$_{10\%}$) in hexafluoro benzene and $CDCl_3$. Incorporated amount of FA/StMA/HEMA is 51.1/40.8/8.1.

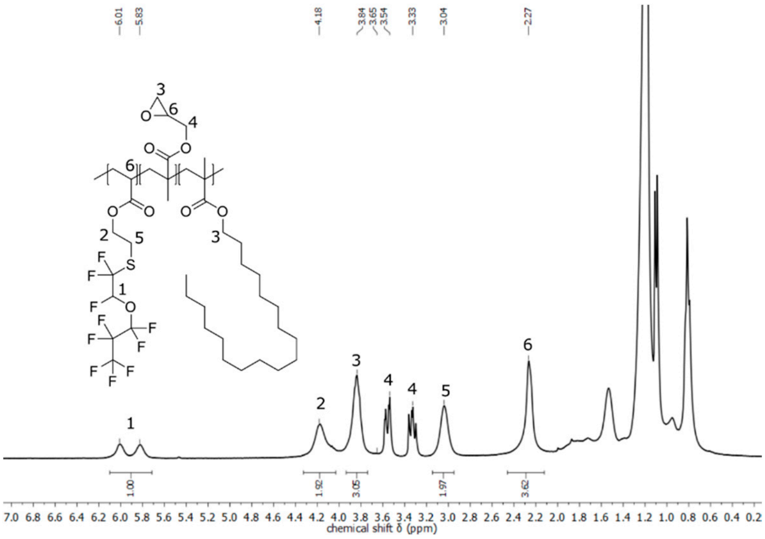

Figure S5. ^{1}H-NMR of P(FA-*co*-StMA-*co*-GlyMA$_{10\%}$) in hexafluoro benzene and $CDCl_3$.

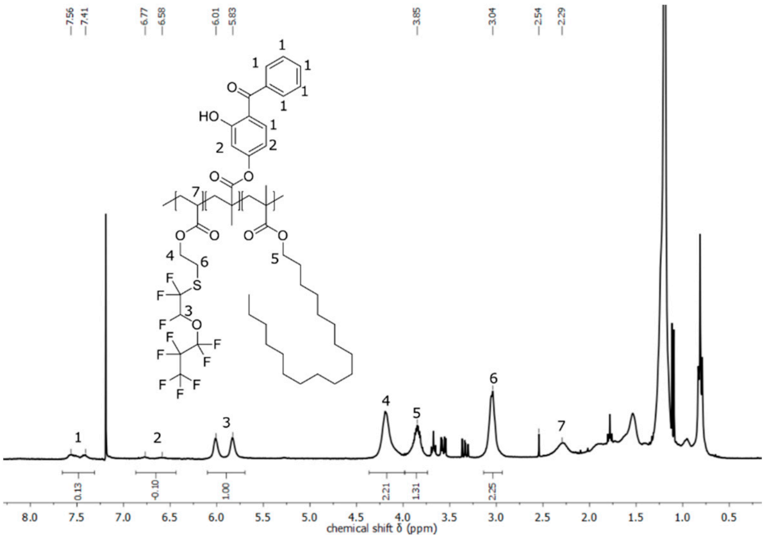

Figure S6. ^{1}H-NMR of P(FA-*co*-StMA-*co*-BPMA$_{2\%}$) in hexafluoro benzene and $CDCl_3$. Incorporated amount of FA/StMA/BPMA is 62.2/36.2/1.6.

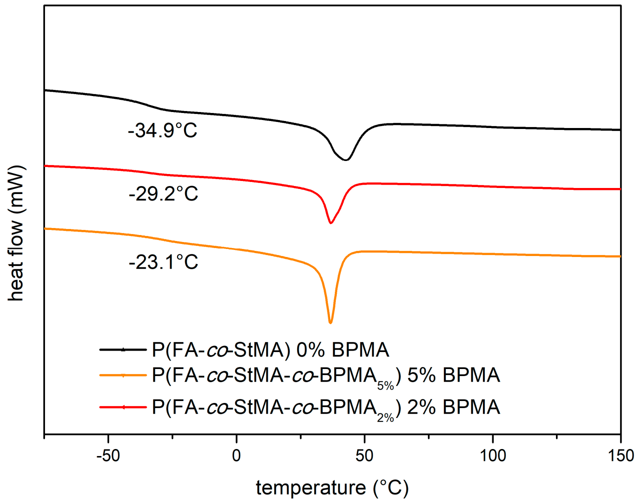

Figure S7. DSC measurements of P(FA-*co*-StMA) (black), P(FA-*co*-StMA-*co*-BPMA2%)(red), and P(FA-*co*-StMA-*co*-BPMA5%) (orange).

Table S1. List of mass increase of the paper coated with different polymers.

	Polymer	Mass Pure Paper (mg)	Mass of Coated Paper (mg)	Increase of Paper Weight (%)
1	P(FA-*co*-StMA)	572.9	609.2	6.33
2	P(FA-*co*-StMA-*co*-HEMA2%) + Crelan EF403	3500.1	3793.6	8.38
3	P(FA-*co*-StMA-*co*-HEMA5%) + Crelan EF403	3532.6	3795.8	7.45
4	P(FA-*co*-StMA-*co*-HEMA10%) + Crelan EF403	3454.6	3750.6	8.56
2	P(FA-*co*-StMA-*co*-HEMA2%) + HMDI	3483.6	3804.8	9.22
3	P(FA-*co*-StMA-*co*-HEMA5%) + HMDI	3510.6	3789.3	7.93
4	P(FA-*co*-StMA-*co*-HEMA10%) + HMDI	3440.9	3727.0	8.31
5	P(FA-*co*-StMA-*co*-GlyMA2%)	569.3	598.1	5.06
6	P(FA-*co*-StMA-*co*-GlyMA5%)	564.9	592.2	4.83
7	P(FA-*co*-StMA-*co*-GlyMA10%)	574.3	598.1	4.14
8	P(FA-*co*-StMA-*co*-BPMA2%)	3546.2	3853.6	8.66
9	P(FA-*co*-StMA-*co*-BPMA5%)	3644.2	3944.8	8.24

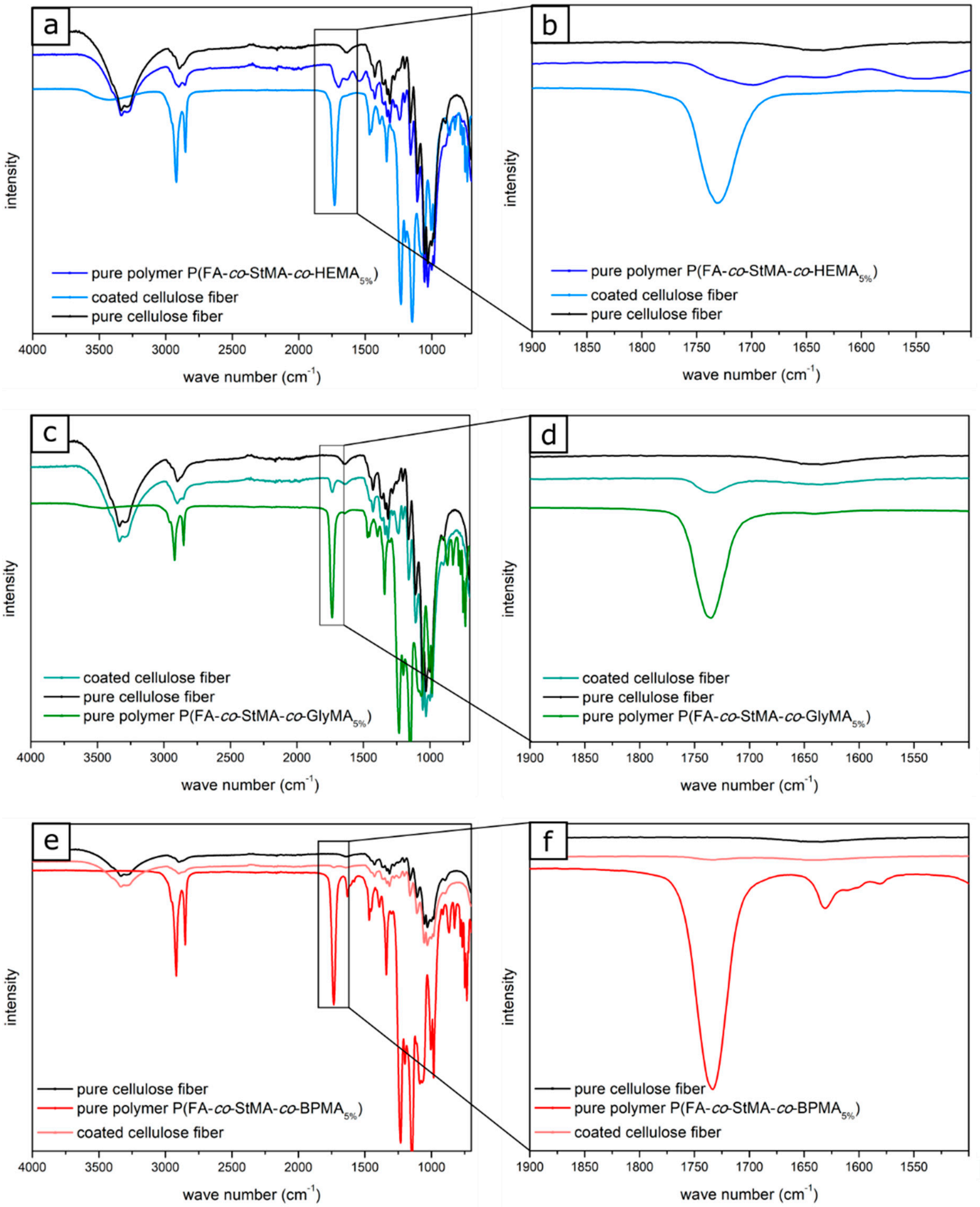

Figure S8. IR spectra of pure polymers, pure cellulose fiber, and coated fiber, and a magnification of the range at 1700 cm^{-1}. **a,b**) P(FA-*co*-StMA-*co*-HEMA$_{5\%}$), **c,d**) P(FA-*co*-StMA-*co*-GlyMA$_{5\%}$), **e,f**) P(FA-*co*-StMA-*co*-BPMA$_{5\%}$).

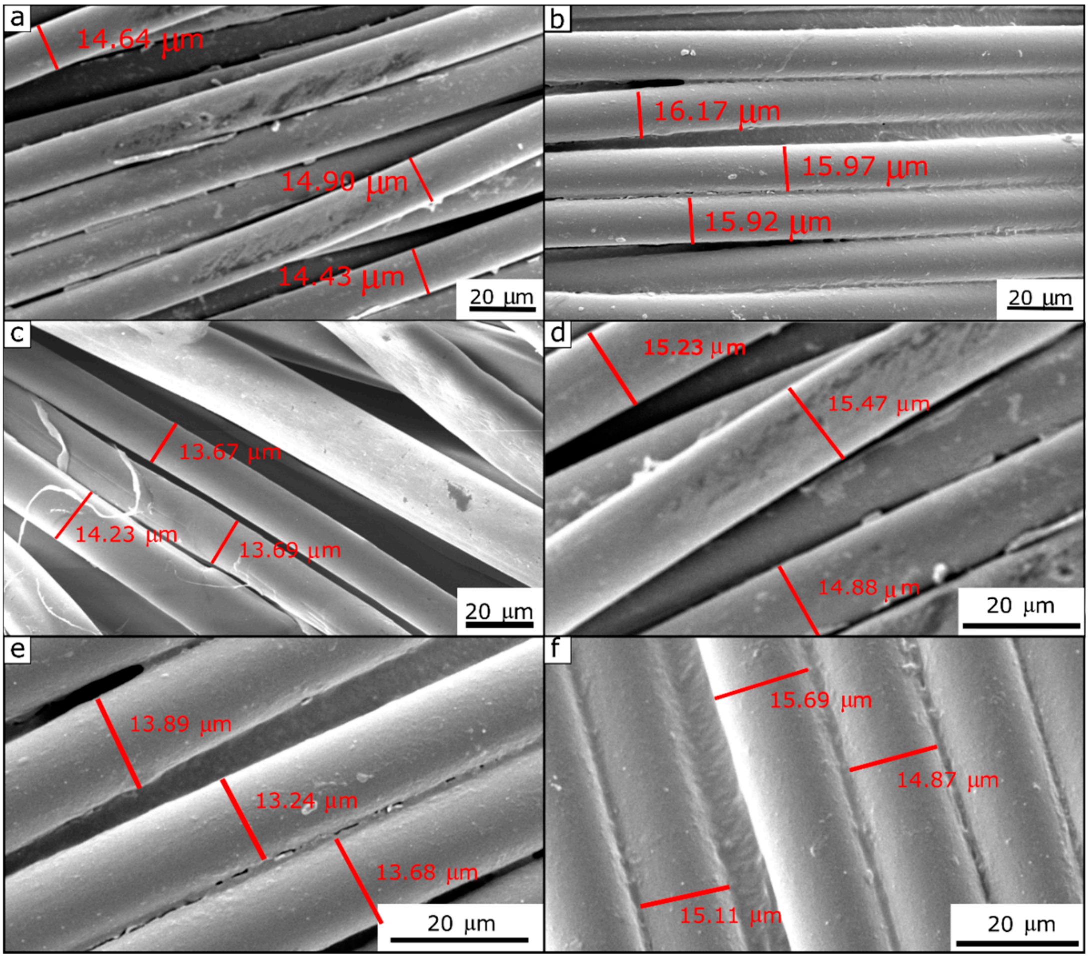

Figure S9. SEM images of PA fibers uncoated (**a,c,,e**) and coated with **b**) P(FA-*co*-StMA-*co*-HEMA$_{5\%}$), **d**) P(FA-*co*-StMA-*co*-GlyMA$_{5\%}$), and **f**) P(FA-*co*-StMA-*co*-BPMA$_{5\%}$). The average increase of fiber diameter is listed in S-11.

Table S2. List of fiber diameters of polyamide fibers in the uncoated state and coated with polymer, as well as the increase in diameter due to coating.

Polymer	Pure PA Fiber Diameter (µm)	Coated Fiber Diameter (µm)	Increase of Diameter (µm)
P(FA-*co*-StMA-*co*-HEMA$_{5\%}$)	14.66	16.02	1.36
P(FA-*co*-StMA-*co*-GlyMA$_{5\%}$)	13.86	15.19	1.33
P(FA-*co*-StMA-*co*-BPMA$_{5\%}$)	13.60	15.22	1.62

Table S3. Test liquids, oil repellency grades, and surface tension of the test liquids of the hydrocarbon test of oil repellency.

Oil Repellency Grade	Test Liquid	Surface Tension (mN/m)
1	kaydol	31.5
2	kaydol:*n*-hexadecane	-
3	*n*-hexadecane	27.3
4	*n*-tetradecane	26.4
5	*n*-dodecane	24.7
6	*n*-decane	23.5
7	*n*-octane	21.8
8	*n*-heptane	20.3

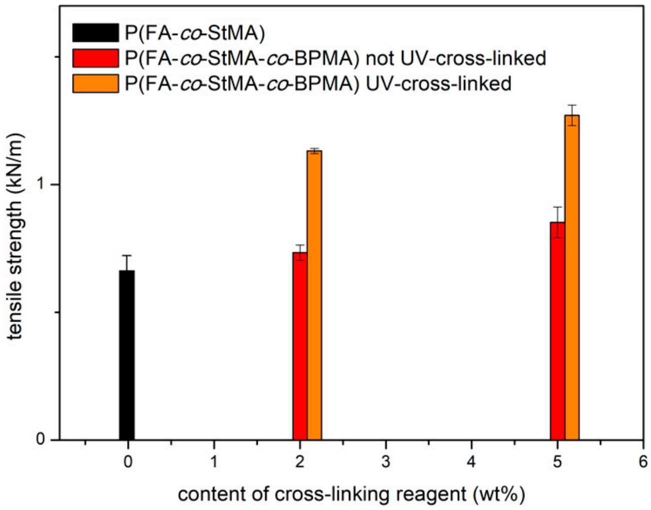

Figure S10. Comparison of the tensile strength of P(FA-*co*-StMA-*co*-BPMA5%) crosslinked with UV irradiation (orange) and not cross linked (red).

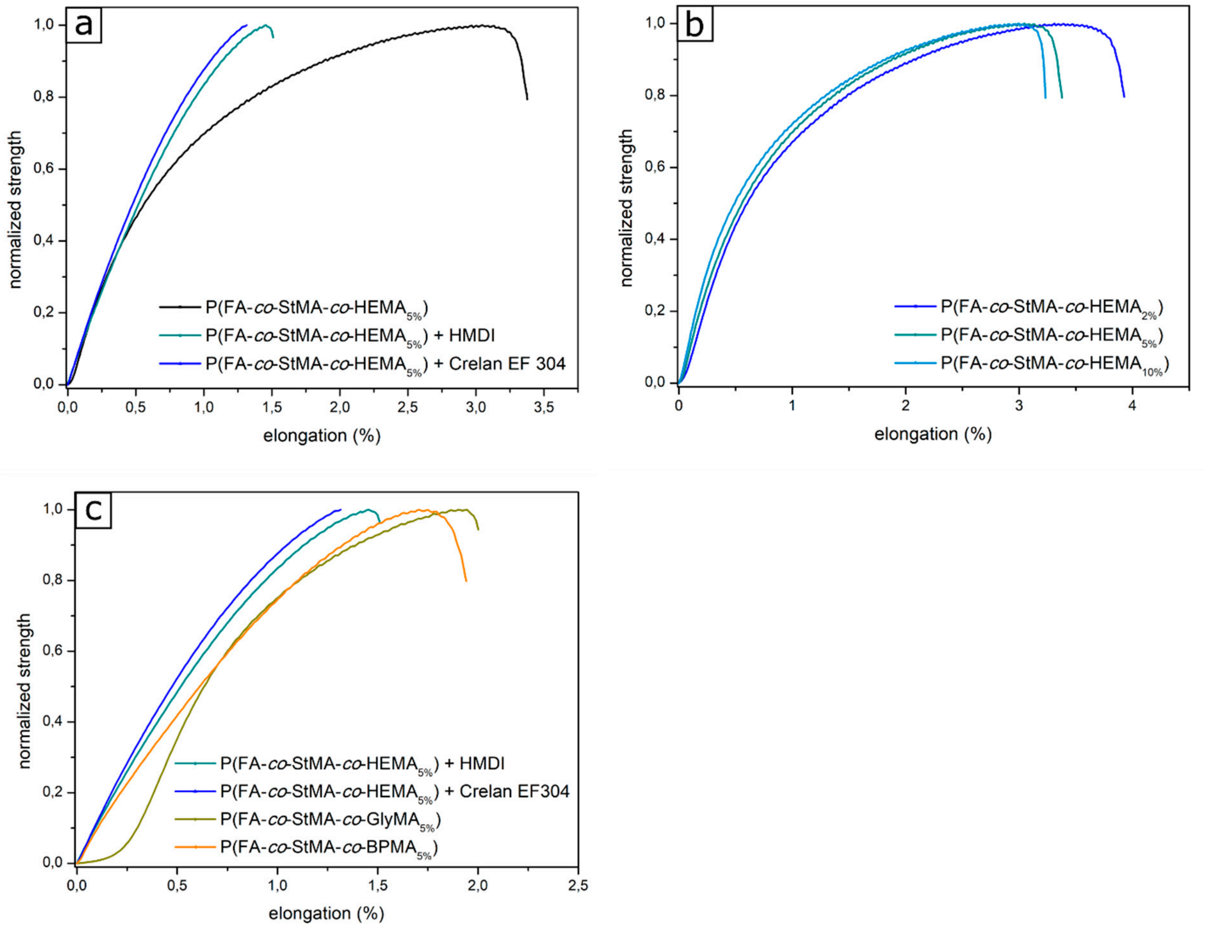

Figure S11. Comparison of the elongation of different polymers. **a**) Comparison of P(FA-*co*-StMA-*co*-HEMA5%) crosslinked with Crelan EF304 or HMDI, **b**) comparison of P(FA-*co*-StMA-*co*-HEMA) with

different cross-linking degrees, and **c**) comparison of P(FA-*co*-StMA-*co*-HEMA$_{5\%}$) with P(FA-*co*-StMA-*co*-GlyMA$_{5\%}$) and P(FA-*co*-StMA-*co*-BPMA$_{5\%}$).

Table S4. Weight loss of polymer through the extraction of fibers with THF.

Polymer	Uncoated Fiber (mg)	Coated Fiber (mg)	Extracted Fiber (mg)	Weight Loss (%)
P(FA-*co*-StMA)	572.9	609.2	577.5	–87.2
P(FA-*co*-StMA-*co*-HEMA$_{2\%}$) + Crelan EF403	367.4	398.1	391.1	–22.8
P(FA-*co*-StMA-*co*-HEMA$_{5\%}$) + Crelan EF403	263.1	337.3	324.6	–17.1
P(FA-*co*-StMA-*co*-HEMA$_{10\%}$) + Crelan EF403	430.3	467.5	460.8	–18.0
P(FA-*co*-StMA-*co*-HEMA$_{2\%}$) + HMDI	453.5	495.3	490.7	–11.0
P(FA-*co*-StMA-*co*-HEMA$_{5\%}$) + HMDI	251.3	273.9	271.5	–10.6
P(FA-*co*-StMA-*co*-HEMA$_{10\%}$) + HMDI	307.9	333.6	332.8	–3.1
P(FA-*co*-StMA-*co*-GlyMA$_{2\%}$)	569.3	598.1	587.7	–36.1
P(FA-*co*-StMA-*co*-GlyMA$_{5\%}$)	564.9	592.2	591.3	–3.3
P(FA-*co*-StMA-*co*-GlyMA$_{10\%}$)	574.3	598.1	597.9	–0.8
P(FA-*co*-StMA-*co*-BPMA$_{2\%}$)	559.6	625.0	594.6	–46.5
P(FA-*co*-StMA-*co*-BPMA$_{5\%}$)	623.5	673.4	660.3	–26.2

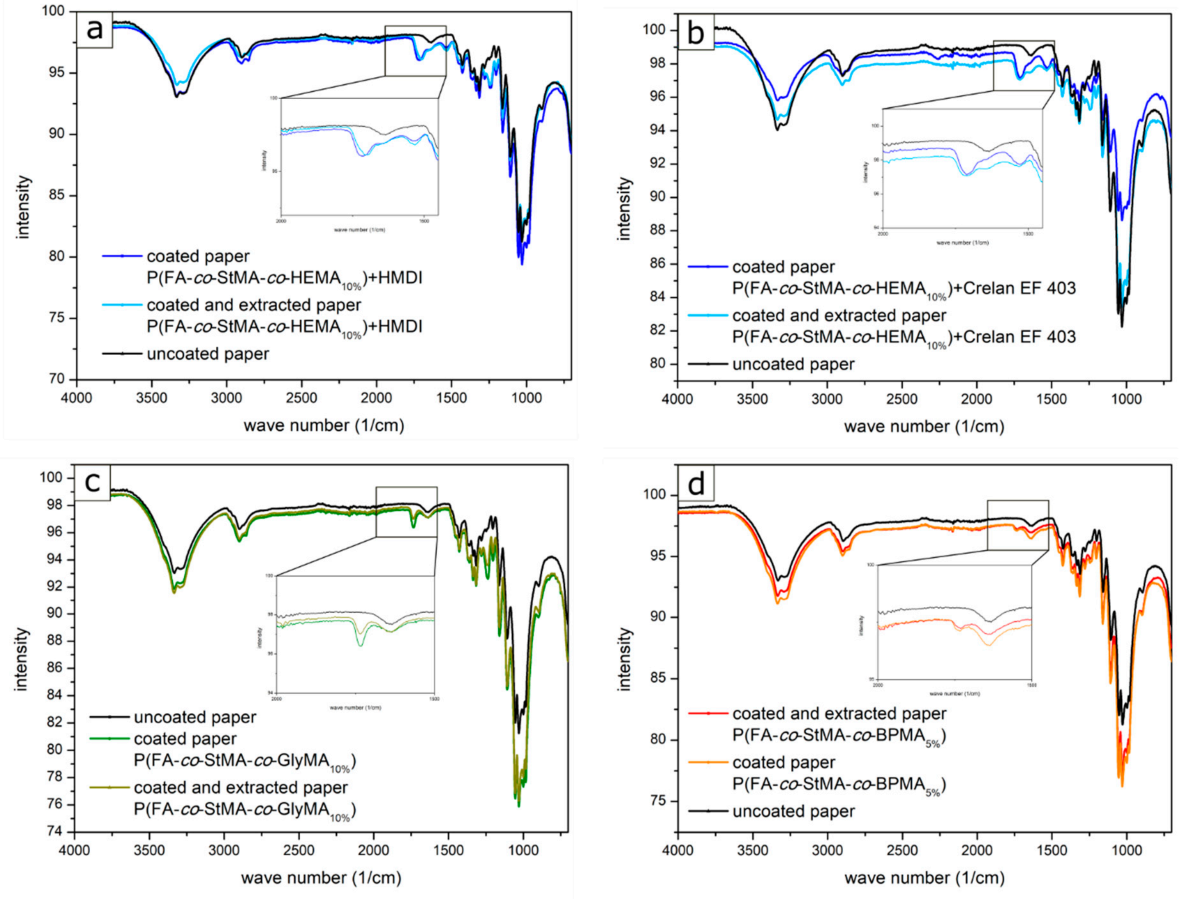

Figure S12. IR spectra of uncoated paper, coated papers, and extracted papers with a magnification in the area at 1700 cm^{-1}. **a**) P(FA-*co*-StMA-*co*-HEMA$_{10\%}$) cross-linked with HMDI, **b**) P(FA-*co*-StMA-*co*-HEMA$_{10\%}$) cross-linked with Crelan EF304, **c**) P(FA-*co*-StMA-*co*-GlyMA$_{10\%}$), and **d**) P(FA-*co*-StMA-*co*-BPMA$_{5\%}$).

Figure S13. Synthesis of Rohdamin B meth arylamide (RhBMA).

For the confocal microscope images, a fluorescent dye monomer, rhodamine B meth acrylamide was prepared as described in literature protocols.

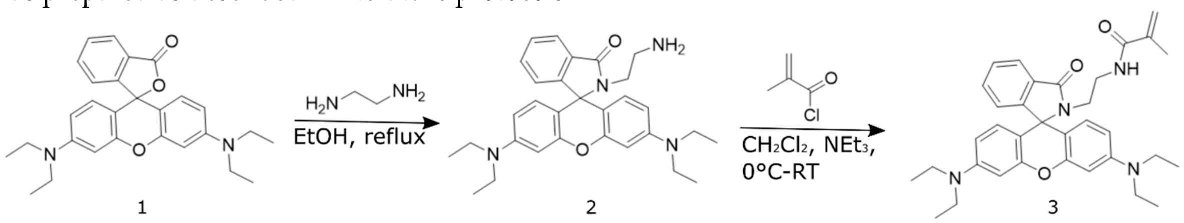

The fluorescent pigment rhodamine B meth acrylamide (3) is produced from rhodamine B base (1) in two steps. In the first step, rhodamine B amine (2) is synthesized from (1) by dissolving 2.4 g (5.4 mmol) (1) in 15 mL ethanol and adding 2.5 mL ethylendiamine dropwise. The solution is stirred for further 18 h under reflux. After cooling to room temperature, the solvent is distilled off under reduced pressure. By adding 15 mL of water to the solid and extracting the mixture twice with 20 mL of dichloromethane byproducts are removed. The combined organic phases are washed twice more with water and dried with sodium sulphate. The solvent is removed and the product rhodamine B amine (2) is dried under vacuum. In total, 1.9 g of a pink solid are formed with a yield of 74%.

To prepare rhodamine B meth arylamide (3), dissolve 0.6 g (1.2 mmol) of rhodamine B amine (2) in 4.4 mL of dichloromethane and add 0.42 mL (4.6 mmol) of trimethylamine under stirring and a stream of nitrogen. A solution of 0.28 mL (2.9 mmol) methacrylic acid chloride in 1.5 mL dichloromethane is then added dropwise at 0 °C. After dosing, the solution is heated to room temperature and stirred for a further 18 h. The mixture is washed eight times with 15 mL of water and three times with 15 mL of saturated sodium carbonate solution and once with saturated sodium chloride solution, dried over magnesium sulphate, and the solvent is distilled of. The result is 0.29 g of a pink solid with a yield of 43%. A ^{1}H-NMR spectrum was recorded for identification and purity determination. The spectrum is shown in Figure S-16 :^{1}H-NMR (300 MHz, $CDCl_3$, TMS) δ(ppm): 7.85 (m, $1H_1$), 7.57 (s, $1H_2$), 7.39 (m, $1H_3$), 7.36 (m, $1H_4$), 7.01 (m, $1H_5$), 6.38 (m, $2H_6$), 6.35 (m, $2H_7$), 6.22 (m, $2H_8$), 5.70, 5.23 (d, $2H_9$), 3.28 (m, $8H_{10}$), 3.25 (m, $2H_{11}$), 2.98 (m, $2H_{12}$) 1.97 (s, $3H_{13}$), 1.19 (m, $12H_{14}$).

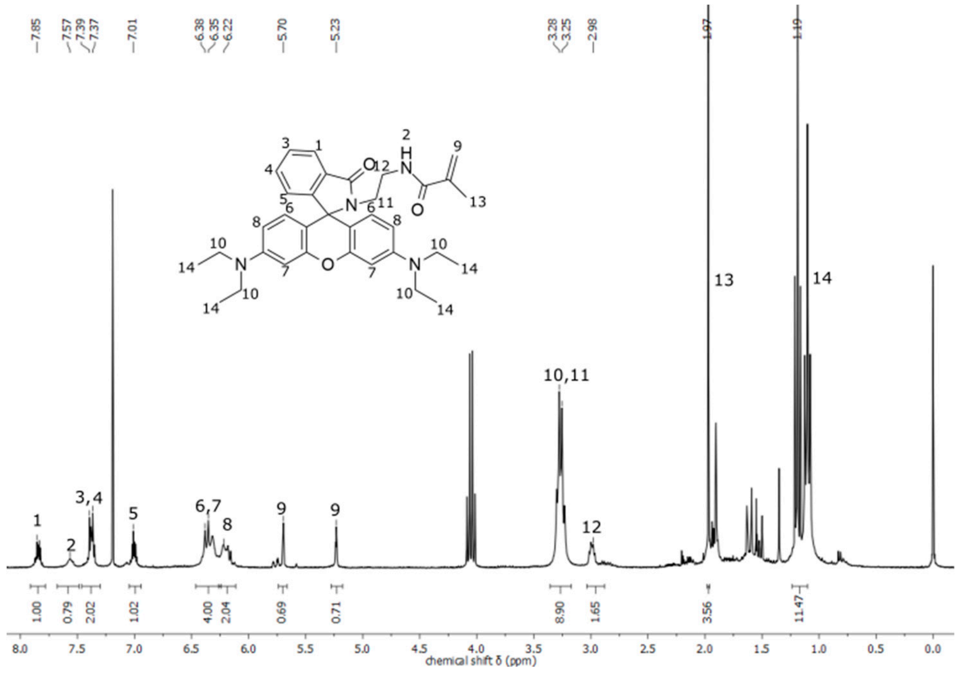

Figure S14. ^{1}H-NMR measurement of rhodamine B meth acrylamide.
